# Supplementary material for: DogCatcher allows loop-friendly protein-protein ligation
Source: Cell Chem Biol. 2022 Feb 17;29(2):339–350.e10. doi: 10.1016/j.chembiol.2021.07.005 (PMC8878318; doi:10.1016/j.chembiol.2021.07.005)
Supplement: Document S1. Figures S1–S6 and Table S1 [file mmc1.pdf]

Cell Chemical Biology, Volume *29*

Supplemental information

DogCatcher allows loop-friendly protein-protein ligation

Anthony H. Keeble, Vikash K. Yadav, Matteo P. Ferla, Claudia C. Bauer, Eulashini Chuntharpursat-Bon, Jin Huang, Robin S. Bon, and Mark Howarth

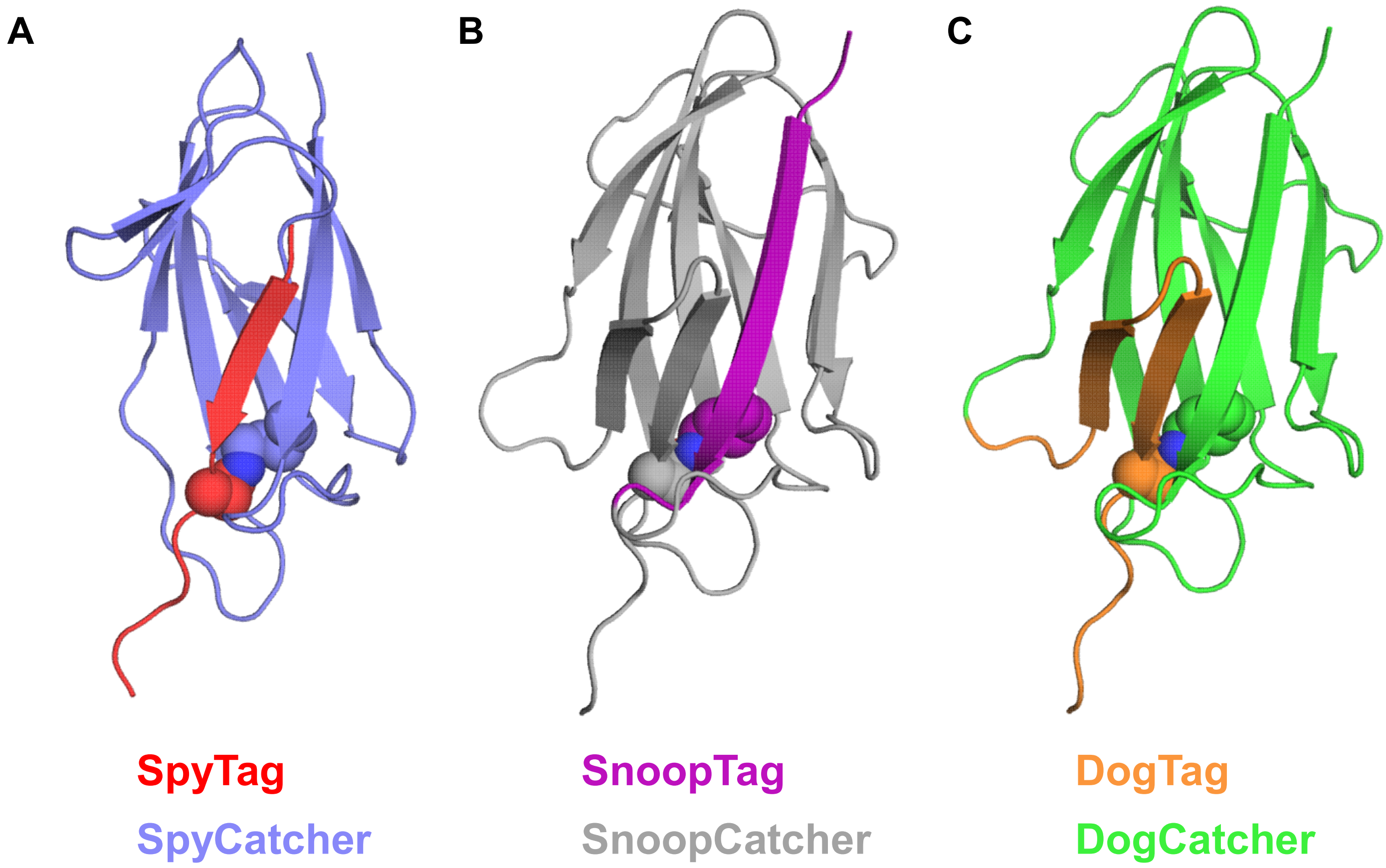

Figure S1: Structural comparison of the different Tag/Catcher pairs, related to Figure 1. **(A)** SpyTag/SpyCatcher has the Tag as a linear single β-strand (PDB: 2X5P and 4MLI). The isopeptide-forming residues of SpyTag (Asp117) and SpyCatcher (Lys31) are shown as spheres. **(B)** Model of SnoopTag/SnoopCatcher based on RrgA domain 4 (PDB: 2WW8). SnoopTag has the Tag derived from a linear single β-strand. The isopeptide-forming residues of SnoopTag (Lys742) and SnoopCatcher (Asn854) are shown as spheres. **(C)** Model of DogTag/DogCatcher based on RrgA domain 4 (PDB: 2WW8). DogTag/DogCatcher has the Tag derived from two β-strands arranged as a β-hairpin. The side-chains for the isopeptide-forming residues of the DogTag (Asn854) and DogCatcher (Lys742) are shown as spheres. The dark blue sphere indicates the nitrogen atom in the isopeptide bond.

A

```
              734   740        750        760        770        780
R2Catcher     KLGDIEFIKVNKNDKKPLRGAVFSLQKQHPDYPDIYGAIDQNGTYQNVRTGEDGK
DogCatcher    KLGEIEFIKVDKTDKKPLRGAVFSLQKQHPDYPDIYGAIDQNGTYQDVRTGEDGK

               790        800        810        820        830
R2Catcher     LTFKNLSDGKYRLFENSEPAGYKPVQNKPIVAFQIVNGEVRDVTSIVPQ-
DogCatcher    LTFTNLSDGKYRLIENSEPPGYKPVQNKPIVSFRIVDGEVRDVTSIVPQ-
```

Reactive Lysine
Catalytic Glutamate
Mutations to create DogCatcher

B

```
                840        850        860
R2Tag         DIPAGYEFTNDKHYITNEPIPPK
DogTag        DIPATYEFTDGKHYITNEPIPPK
```

Reactive Asparagine
Mutations to create DogTag

C

```
              734   740        750        760        770        780
Domain 4      KLGDIEFIKVNKNDKKPLRGAVFSLQKQHPDYPDIYGAIDQNGTYQNVRTGEDGK
SnoopCatcher               NDKKPLRGAVFSLQKQHPDYPDIYGAIDQNGTYQNVRTGEDGK
SnoopLigase             VNKNDKKPLRGAVFSLQKQHPDYPDIYGAIDQNGTYQNVRTGEDGK
DogCatcher    KLGEIEFIKVDKTDKKPLRGAVFSLQKQHPDYPDIYGAIDQNGTYQDVRTGEDGK

               790        800        810        820        830        840
Domain 4      LTFKNLSDGKYRLFENSEPAGYKPVQNKPIVAFQIVNGEVRDVTSIVPQDIPAGY
SnoopCatcher  LTFKNLSDGKYRLFENSEPAGYKPVQNKPIVAFQIVNGEVRDVTSIVPQDIPATY
SnoopLigase   LTFKNLSDGKYRLFENSEPPGYKPVQNKPIVAFQIVNGEVRDVTSIVPPGVPATY
DogCatcher    LTFTNLSDGKYRLIENSEPPGYKPVQNKPIVSFRIVDGEVRDVTSIVPQ-

                    850        860
Domain4       EFTNDKHYITNEPIPPK-
SnoopCatcher  EFTNGKHYITNEPIPPK-
SnoopLigase   EFT-
DogCatcher
```

Reactive Lysine
Catalytic Glutamate
Reactive Asparagine

Figure S2: Amino acid sequences of the key protein variants, related to Figure 1. (A) Sequence alignment of R2Catcher with DogCatcher. **(B)** Sequence alignment of R2Tag with DogTag. **(C)** Sequence alignments of the ways that RrgA domain 4 has been split to create different protein coupling reagents. Numbering is based on PDB 2WW8.

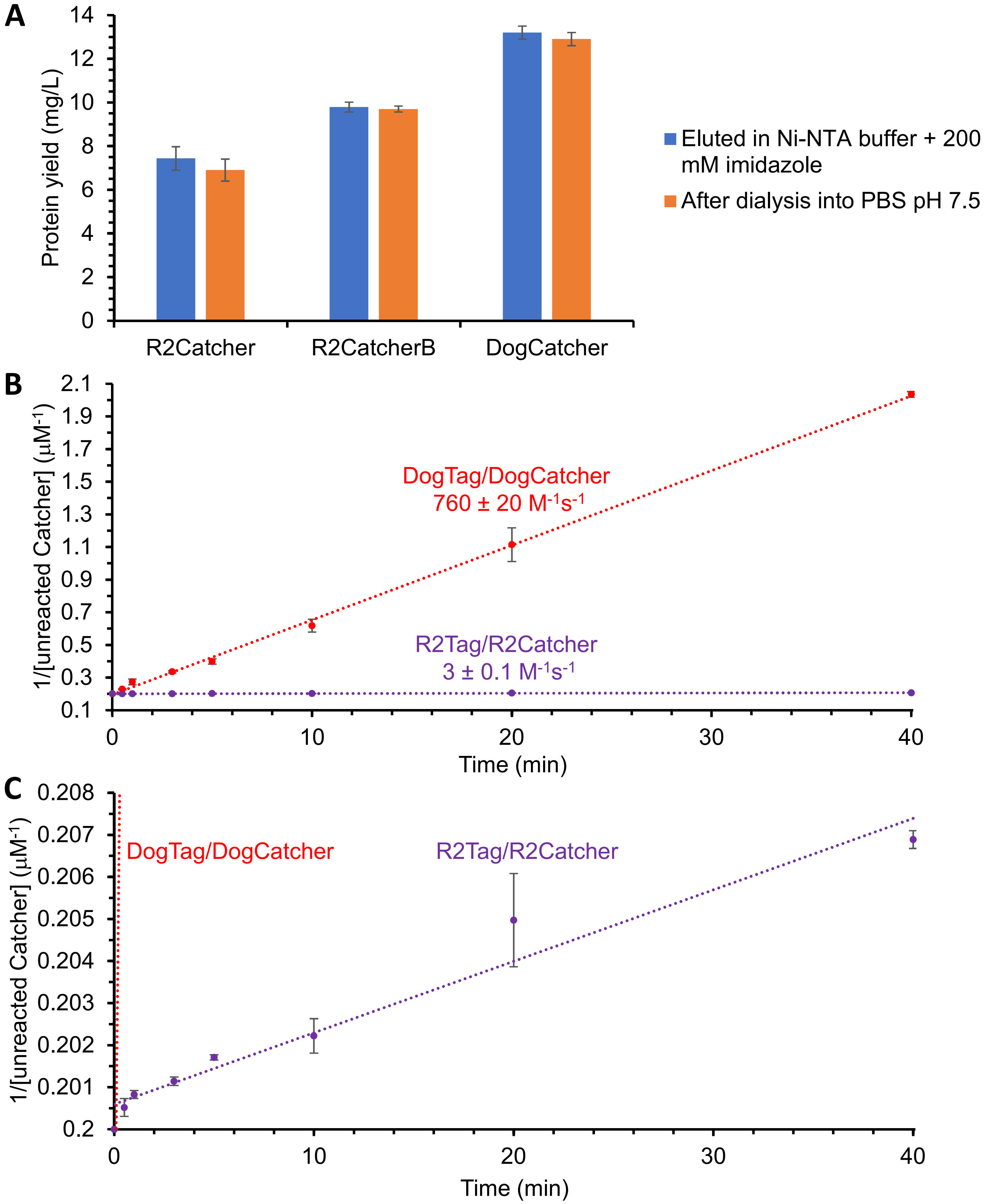

Figure S3: Comparison of solubility and reaction rate, related to Figures 1 and 5. (A) Yield of soluble protein for Catcher variants per liter of *E. coli* culture, determined after Ni-NTA elution (blue) or after dialysis (orange) (mean of duplicate with error bars signifying variation between measurements). **(B)** Time-course of reaction for DogTag/DogCatcher or R2Tag/R2Catcher. 5 μM AviTag-DogTag-MBP and 5 μM DogCatcher or 5 μM AviTag-R2Tag-MBP and 5 μM R2Catcher were incubated in PBS pH 7.5 at 25 °C, with quantification by SDS-PAGE/Coomassie (mean ± 1 s.d., n=3). Some error bars are too small to be visible. The resultant second-order rate constant is marked (mean ± 1 s.d., n=3). **(C)** Zoom of the y-axis from (B), to make the data clearer for R2Tag/R2Catcher.

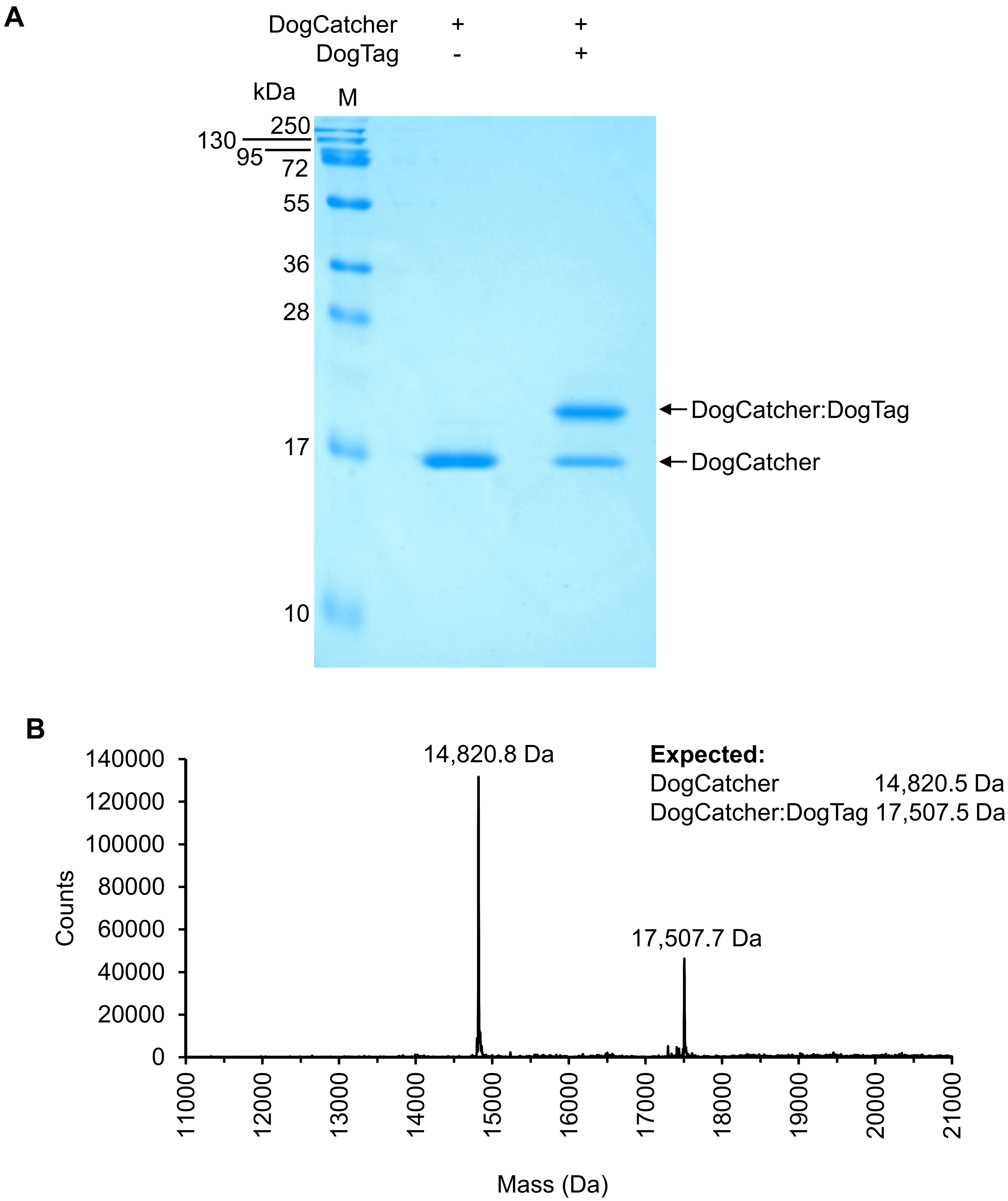

Figure S4: Electrospray ionization mass spectrometry validation of DogCatcher and reaction with DogTag, related to Figure 1. (A) SDS-PAGE with Coomassie staining, illustrating reaction of DogCatcher with DogTag peptide to approximately 50%, so that pre- and post-reacted DogCatcher can be compared. **(B)** Electrospray ionization mass spectrometry of DogCatcher and the reaction product of DogCatcher with DogTag peptide. Expected mass for DogTag peptide + DogCatcher = 17,524.5 Da minus 17 Da (NH_3 released upon isopeptide bond formation) = 17,507.5 Da.

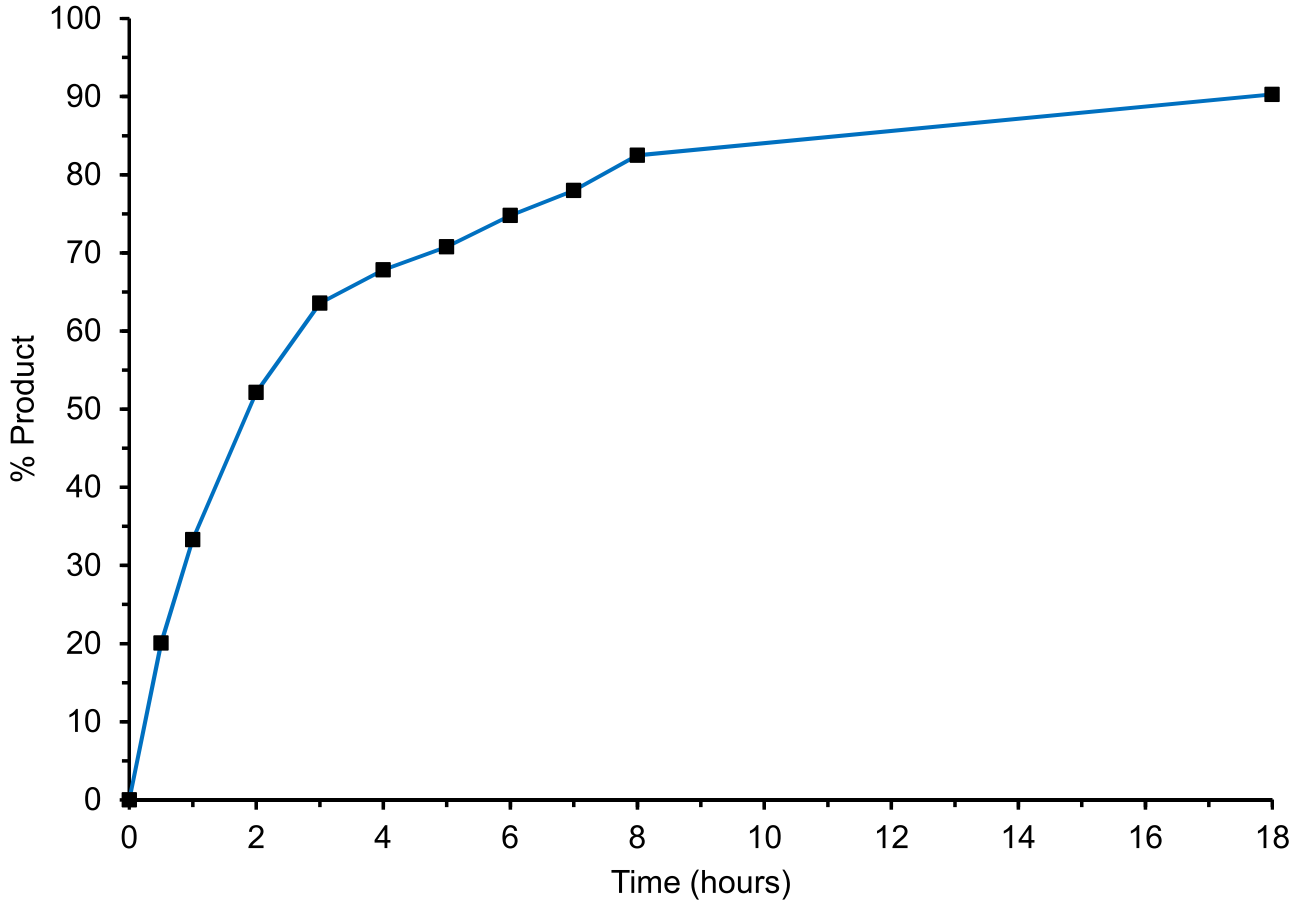

Figure S5: DogTag/DogCatcher reacted efficiently at nanomolar concentrations, related to Figure 3. Time-course of reaction for 100 nM HaloTag7SS-DogTag with 100 nM DogCatcher-sfGFP in PBS pH 7.5 + 0.2% (w/v) BSA at 25 ^{o}C, with quantification by SDS-PAGE/Coomassie and densitometry.

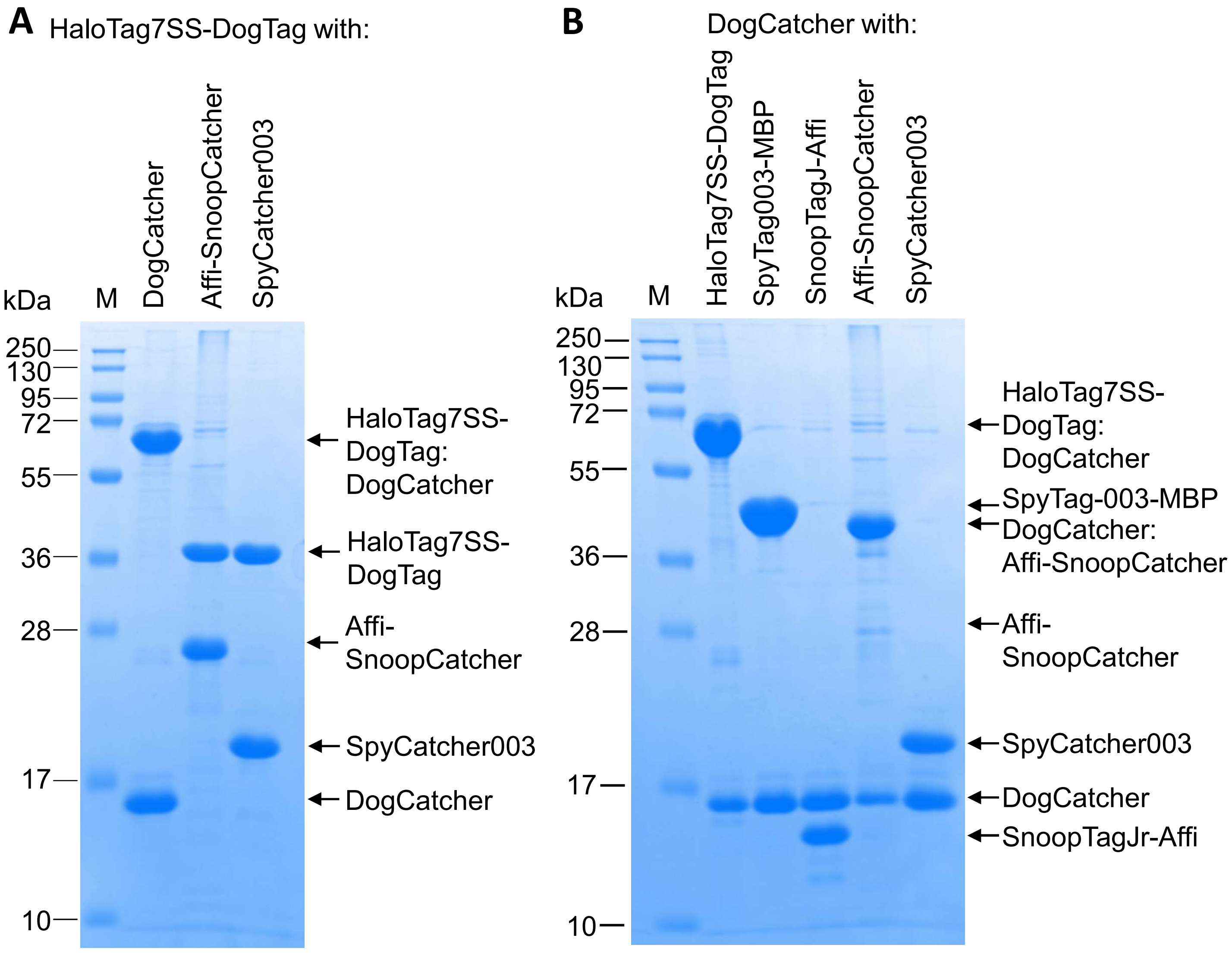

Figure S6: DogTag/DogCatcher orthogonality, as described in STAR Methods (Isopeptide Bond Formation Assays). (**A**) DogTag reacted with DogCatcher but not SnoopCatcher or SpyCatcher003. 15 μM DogCatcher, Affi-SnoopCatcher or SpyCatcher003 was incubated with 10 μM HaloTag7SS-DogTag for 24 h in PBS pH 7.5 at 25 °C, before SDS-PAGE with Coomassie staining. (**B**) DogCatcher reacted with DogTag and SnoopCatcher. 15 μM DogCatcher was incubated with 10 μM HaloTag7SS-DogTag, SpyTag003-MBP, SnoopTagJr-Affi, Affi-SnoopCatcher or SpyCatcher003 for 24 h in PBS pH 7.5 at 25 °C, before SDS-PAGE with Coomassie staining. M = molecular weight markers.

Gre2p Variant	**Specific activity ($\mu mol_{NAPDH}.min^{-1}.\mu mol_{protein}^{-1}$)**
WT	1,892 ± 294
SpyTag003 Loop A	2,391 ± 347
SpyTag003 Loop B	1,572 ± 372
SpyTag003 Loop C	1,814 ± 83
DogTag Loop A	3,087 ± 259
DogTag Loop B	3,268 ± 361
DogTag Loop C	1,484 ± 223

Table S1: Specific enzyme activities for Gre2p variants, related to Figure 6. Each Gre2p variant was incubated with isovaleraldehyde and NADPH in phosphate buffer at 25 ^{o}C and reaction was monitored spectrophotometrically (mean ± 1 s.d., n=3 biological replicates).
