# Supplementary material for: DogCatcher allows loop-friendly protein-protein ligation
Source: Cell Chem Biol. 2022 Feb 17;29(2):339–350.e10. doi: 10.1016/j.chembiol.2021.07.005 (PMC8878318; doi:10.1016/j.chembiol.2021.07.005)
Supplement: Document S2. Article plus supplemental information [file mmc2.pdf]

Resource

Cell Chemical Biology

DogCatcher allows loop-friendly protein-protein ligation

Graphical abstract

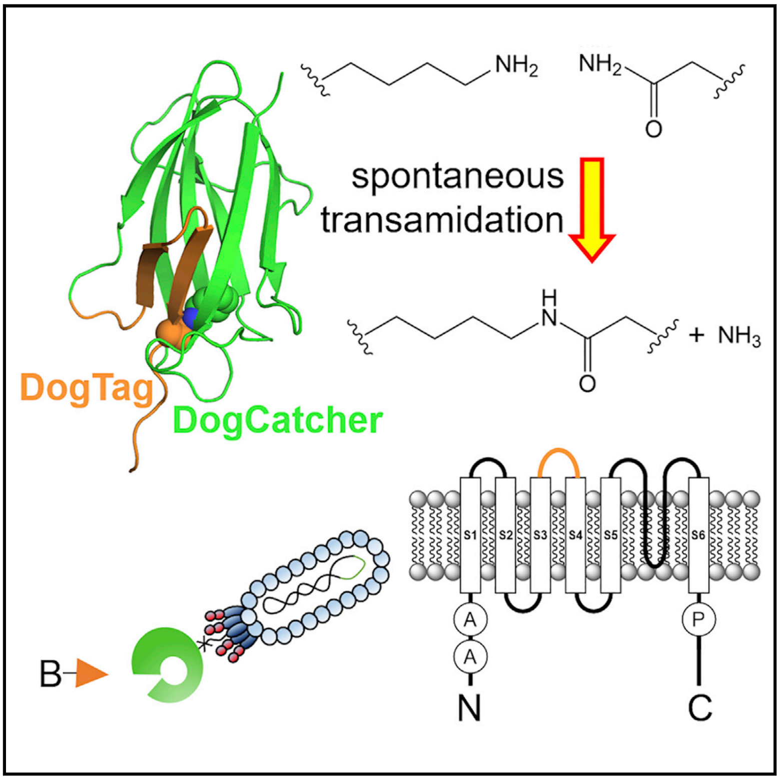

Highlights

- Spontaneous transamidation at internal sites harnessing a DogTag/DogCatcher pair
- DogCatcher is designed and bred for high solubility and rapid reaction
- Within protein loops DogTag can clamp on its partner faster than SpyTag003
- Fast and faithful fluorescent labeling of an ion channel at the cell surface via DogTag

Authors

Anthony H. Keeble, Vikash K. Yadav, Matteo P. Ferla, ..., Jin Huang, Robin S. Bon, Mark Howarth

Correspondence

mark.howarth@bioch.ox.ac.uk

In brief

Loopophilic protein coupling is achieved here by re-engineering a pathogenic adhesion protein. Many conjugation approaches only function at exposed termini. Keeble et al. create DogTag/DogCatcher for high-yield covalent labeling. Reactivity is robust to conditions and maintains protein function for catalysis, fluorescence and ion conductance after DogTag loop insertion.

Keeble et al., 2022, Cell Chemical Biology 29, 339–350
February 17, 2022 © 2021 The Authors. Published by Elsevier Ltd.
https://doi.org/10.1016/j.chembiol.2021.07.005

CellPress

Cell Chemical Biology

CellPress
OPEN ACCESS

Resource

DogCatcher allows loop-friendly protein-protein ligation

Anthony H. Keeble,[1] Vikash K. Yadav,[1] Matteo P. Ferla,[1,3] Claudia C. Bauer,[2] Eulashini Chuntharpursat-Bon,[2] Jin Huang,[1] Robin S. Bon,[2] and Mark Howarth[1,4,*]

[1]Department of Biochemistry, University of Oxford, South Parks Road, Oxford OX1 3QU, UK

[2]Discovery and Translational Science Department, Leeds Institute of Cardiovascular and Metabolic Medicine, School of Medicine, University of Leeds, Leeds LS2 9JT, UK

[3]Present address: Wellcome Center for Human Genetics, University of Oxford, Roosevelt Drive, Headington, Oxford OX3 7BN, UK.

[4]Lead contact

*Correspondence: mark.howarth@bioch.ox.ac.uk

https://doi.org/10.1016/j.chembiol.2021.07.005

SUMMARY

There are many efficient ways to connect proteins at termini. However, connecting at a loop is difficult because of lower flexibility and variable environment. Here, we have developed DogCatcher, a protein that forms a spontaneous isopeptide bond with DogTag peptide. DogTag/DogCatcher was generated initially by splitting a *Streptococcus pneumoniae* adhesin. We optimized DogTag/DogCatcher through rational design and evolution, increasing reaction rate by 250-fold and establishing millimolar solubility of DogCatcher. When fused to a protein terminus, DogTag/DogCatcher reacts slower than SpyTag003/SpyCatcher003. However, inserted in loops of a fluorescent protein or enzyme, DogTag reacts much faster than SpyTag003. Like many membrane proteins, the ion channel TRPC5 has no surface-exposed termini. DogTag in a TRPC5 extracellular loop allowed normal calcium flux and specific covalent labeling on cells in 1 min. DogTag/DogCatcher reacts under diverse conditions, at nanomolar concentrations, and to 98% conversion. Loop-friendly ligation should expand the toolbox for creating protein architectures.

INTRODUCTION

Engineering unnatural protein architectures may contribute to some of society's biggest challenges, such as rapid-response vaccines (Brune and Howarth, 2018; Morris et al., 2019) or enzyme synergy for agricultural productivity (Qu et al., 2019; Sweetlove and Fernie, 2018) and pollutant degradation (Knott et al., 2020). Extensive work has been done to establish post-translational connection of protein units, including native chemical ligation, split inteins, sortase, and butelase (Banerjee and Howarth, 2018). However, there has been much less attention to protein-protein ligation at internal sites, where there is more steric hindrance and fewer accessible chemistries. N and C termini of natural proteins are often highly flexible and more exposed, facilitating reaction (Jacob and Unger, 2007). Internal loops may adopt diverse structures and there are countless examples of insertion of a peptide tag in a loop interfering with protein folding or function (Oesterle et al., 2017). Even antibody recognition of epitope tags in loops is challenging: common epitope tags, such as FLAG tag, do not perform well in protein loops (Fujii et al., 2016). Various cysteine-dependent routes have been used to ligate proteins, but suffer from competing homodimerization, as well as folding complications from pre-existing disulfide bonds (Baneyx and Mujacic, 2004). Recently a sortase from *Corynebacterium diphtheriae* was established for ligation to lysine at internal protein sites but uses millimolar substrate peptide (Sue et al., 2020). Branched proteins can be accessed through protein semi-synthesis, such as with δ-mercaptolysine, requiring coupling to a thioester and then desulfurization (Jbara et al., 2018). Unnatural amino acids allow click chemistry functionalization of proteins at diverse sites, but increase the complexity of protein production (Manandhar et al., 2021). Transglutaminases are also efficient at labeling of internal sites but have challenges with specificity (Schneider et al., 2020). Lysine acylation using conjugating enzymes can label proteins at internal sites, although this elegant approach requires the generation of a thioester substrate for conjugation (Hofmann et al., 2020).

For efficient and genetically encodable protein-protein ligation, we previously developed SpyTag/SpyCatcher, allowing spontaneous isopeptide bond formation to a peptide tag (Figure S1) (Zakeri et al., 2012). Through computational design and directed evolution, our latest pair consists of a peptide (SpyTag003) that reacts through spontaneous amidation with its protein partner (SpyCatcher003) at a rate approaching the diffusion limit (Keeble et al., 2019). More than 800 constructs have been published with SpyTag/SpyCatcher or its relatives at terminal sites on proteins, but there is little study of performance in protein loops (Keeble and Howarth, 2019). Fusion of SpyTag in a loop of the multipass membrane protein Orai1

This is an open access article under the CC BY license (http://creativecommons.org/licenses/by/4.0/).

indicated that SpyCatcher reaction was inefficient in this context (Bae et al., 2021). Probing the utility of SpyTag003 for reaction in protein loops, we show that SpyTag003 reaction with SpyCatcher003 was dramatically slowed within loops. Therefore, here we develop and optimize a reactive pair, DogTag/DogCatcher, specifically focused on efficient reaction within loops. We demonstrate the high reactivity of DogTag/DogCatcher in loops of a fluorescent protein and enzyme. We also establish the use of DogTag/DogCatcher for rapid loop-mediated labeling of ion channels in live mammalian cells.

RESULTS

Alternative splitting of RrgA domain 4 to create a Tag-Catcher pair

RrgA is an adhesin from *S. pneumoniae* that consists of four domains. Domain 4 (residues 734–861) forms a spontaneous intramolecular isopeptide bond by a transamidation reaction between Lys742 and Asn854, facilitated by proton transfer via Glu803 (Figure 1A) (Izore et al., 2010). This domain was previously split and engineered to create the protein-coupling reagents SnoopTag (residues 734–748 containing the reactive Lys742) and SnoopCatcher (residues 749–860 containing Glu803 and the reactive Asn854) (Figure S1 and S2C) (Veggiani et al., 2016). In common with SpyTag/SpyCatcher, SnoopTag adopts a single extended β strand upon reaction with SnoopCatcher (Figure S1) (Li et al., 2014; Veggiani et al., 2016). However, we hypothesized that the β hairpin of domain 4 (residues 839–860, termed R2Tag, Figures S2A and S2B), could be an excellent foundation for a loop-friendly Tag/Catcher. We genetically split the rest of domain 4 of RrgA, giving R2Catcher (residues 734–838 containing the reactive Lys and catalytic Glu) (Figures 1A, 1B, S2A, and S2B).

We found that R2Tag and R2Catcher did successfully reconstitute and react upon mixing, but the rate was slow (Figure 1D). We determined a second-order rate constant of 3 ± 0.1 M^{-1} s^{-1} (mean ± 1 SD, n = 3) in PBS, pH 7.4, at 25°C (Figures S3B and S3C). Building on parallel work in our group on SnoopCatcher and SnoopLigase engineering (Buldun et al., 2018; Veggiani et al., 2016), we first engineered R2Tag for faster reconstitution. The flexible Gly at 842 within a β strand was substituted with Thr, maintaining hydrophilicity and being favored within β sheets. Asp848 was substituted with Gly to favor tight turn formation (Figure 1E). Asn847 was substituted with Asp to improve electrostatic interaction with Lys 849 (Buldun et al., 2018). R2Tag with the mutations G842T, N847D, and D848G (DogTag) improved the reaction by 10-fold with R2Catcher. The second-order rate constant for DogTag with R2Catcher was 30 ± 2 M^{-1} s^{-1} (mean ± 1 SD, n = 3) (Figure 1D).

Rational improvement of Catcher solubility

A major problem for R2Catcher was its limited solubility, when compared with SpyCatcher (>1 mM) (Li et al., 2014). We introduced the A808P mutation to reduce the conformational flexibility of a β turn in R2Catcher (Buldun et al., 2018; Fu et al., 2009; Trevino et al., 2007). We had previously optimized SnoopLigase computationally via PROSS (Goldenzweig et al., 2016) and Rosetta (Leaver-Fay et al., 2011), leading to mutations D737S, D838G, and I839V (Buldun et al., 2018). However, mutation of acidic residues in R2Catcher variants led to highly insoluble proteins at neutral pH. We noticed that the predicted pI of our R2Catcher construct was close to neutral (6.6), compared with 4.5–4.9 for the very soluble SpyCatcher family (Keeble et al., 2017, 2019; Zakeri et al., 2012). Therefore, we introduced four mutations to make the surface charge of R2Catcher more negative: N744D, N780D, K792T, and N825D (Figure 1E). Based on Rosetta prediction for improving fold stability (Leaver-Fay et al., 2011), we also included D737E and N746T (Figure 1E). This variant, termed R2CatcherB, showed increased yield of soluble protein following *E. coli* purification (Figure S3A). We also compared the solubility by spin concentrating until the onset of aggregation: R2Catcher started to aggregate at 0.6 mM, while R2CatcherB could be concentrated to >2 mM without observed aggregation.

Phage display selection of improved Catcher reactivity

Phage display of protein scaffolds for the first time often runs into obstacles, including misfolding, degradation in the periplasm, loss of phage infectivity, and accumulation of frame-shifted or truncated variants (Beekwilder et al., 1999; Hentrich et al., 2021; Steiner et al., 2006). Therefore, with the more soluble R2CatcherB in hand as a starting point for display on phage, we applied directed evolution to enhance reaction speed with DogTag. We generated a library of mutations in R2CatcherB by error-prone PCR (Figure 1C). During conventional phage display panning, non-covalently bound phage are eluted from the bait protein by conditions, such as glycine at pH 2.5. In the current approach, this same wash is used to remove any non-covalently bound phage, to select only for variants that allow isopeptide bond formation to occur. Phage are then specifically eluted using TEV protease (Figure 1C). After testing various library generation strategies and multiple rounds of selection, our best performing variant, termed DogCatcher, reacted with AviTag-DogTag-MBP 25-fold faster than R2Catcher (760 ± 20 M^{-1} s^{-1}, mean ± 1 SD, n = 3) (Figures 1D, S3B, and S3C). DogCatcher contained three further mutations compared with R2CatcherB (F802I, A820S, and Q822R) (Figure S2A), which we illustrate on the structure of the parent domain in Figure 1E. Overall, DogTag/DogCatcher represents a 250-fold improvement of the rate of reaction over the parent split pair (R2Tag and R2Catcher) (Figures 1D, S3B, and S3C). We confirmed the isopeptide bond formation in the DogTag:DogCatcher complex by electrospray ionization mass spectrometry (Figure S4). Soluble expression of DogCatcher was enhanced over R2CatcherB (Figure S3A). We did not detect aggregation of DogCatcher in PBS, pH 7.5, upon spin concentrating until the concentration reached 1.8 mM.

DogTag/DogCatcher had different dependence on conditions to SpyTag003/SpyCatcher003

Having settled on our optimized split pair, DogTag/DogCatcher, we thoroughly characterized its dependence on reaction conditions (Figure 2). In parallel, we also determined the condition dependence of SpyTag003/SpyCatcher003 (Figure 3), which reacts near the diffusion limit under optimal circumstances (Keeble et al., 2019) but has not been characterized under diverse conditions.

DogTag/DogCatcher reacted poorly at pH 4 and 5, with reactivity rising sharply to pH 7 and high reactivity maintained at pH

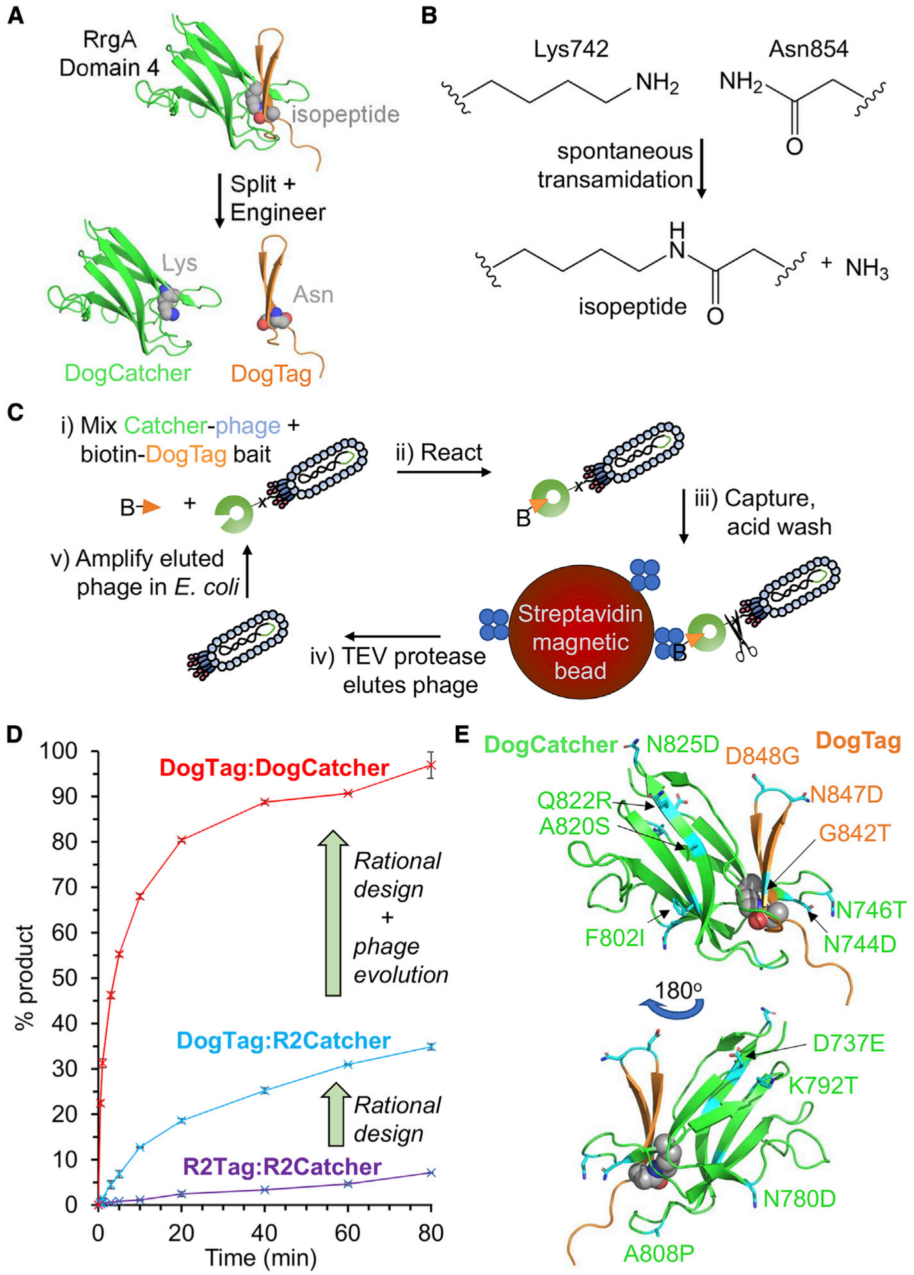

Figure 1. Splitting and engineering to create DogTag/DogCatcher

(A) Domain splitting. RrgA domain 4 contains an intramolecular isopeptide bond (shown as spheres; schematic based on PDB: 2WW8). The domain was genetically split to create R2Catcher and a β hairpin called R2Tag, which, after further engineering, became DogCatcher and DogTag.

(B) Chemistry of amide bond formation between Lys742 of DogCatcher and Asn854 of DogTag.

(C) Phage display evolution of DogCatcher. A library of error-prone Catcher variants was displayed on M13 phage pIII and panned for rapid covalent bond formation to DogTag bait linked to biotin (B).

(D) Enhancement of reaction speed. Amide bond formation rate for R2Tag/R2Catcher (purple trace), DogTag/R2Catcher (cyan trace), or DogTag/DogCatcher (red trace) in PBS (pH 7.5) at 25°C with 5 μM of each protein. Mean ± 1 SD, n = 3 based on SDS-PAGE densitometry. Some error bars are too small to be visible.

(E) Mapping of the mutations (cyan) engineered into RrgA domain 4 (PDB: 2WW8) to create DogCatcher (green) and DogTag (orange). A second view is shown with 180° rotation to illustrate residues on the opposite face.

8 and 9 (Figure 2A). In contrast, SpyTag003/SpyCatcher003 reacted poorly at pH 9, but retained good reactivity from pH 5 to 7 (Figure 3A). The pH dependence of DogTag/DogCatcher was similar to SnoopTag/SnoopCatcher, from the same RrgA domain (Figure S1) (Veggiani et al., 2016). SpyTag003/SpyCatcher003 retained similar pH dependence to the previous SpyTag/SpyCatcher series, being high at pH 7 and peaking at pH 6 (Keeble et al., 2017; Zakeri et al., 2012). Both DogTag/DogCatcher and

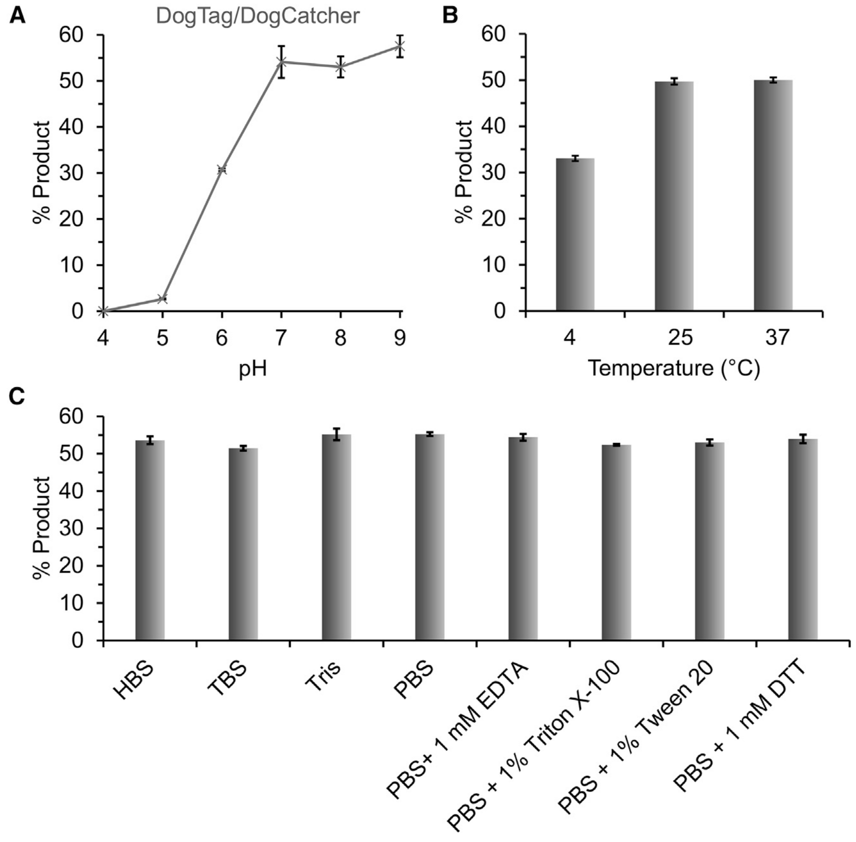

Figure 2. Condition dependence of DogTag/DogCatcher reactivity

(A) pH dependence: 2 μM AviTag-DogTag-MBP and 2 μM DogCatcher were reacted for 30 min at 25°C in SPG buffer at the indicated pH.

(B) Temperature dependence: 2 μM AviTag-DogTag-MBP and 2 μM DogCatcher were reacted for 30 min in SPG, pH 7.0, at the indicated temperature.

(C) Buffer dependence: 5 μM AviTag-DogTag-MBP and 5 μM DogCatcher were reacted for 5 min at 25°C, pH 7.5, in the indicated buffer. HBS, HEPES-buffered saline; TBS, Tris-buffered saline. Mean ± 1 SD, n = 3; some error bars are too small to be visible.

SpyTag003/SpyCatcher had substantial activity at 4°C, along with high reactivity from 25°C to 37°C (Figures 2B and 3B). DogTag/DogCatcher showed high reactivity in a range of buffers (HEPES, PBS, Tris) and was tolerant to chelator (EDTA) or detergent (Figure 2C). SpyTag003/SpyCatcher003 was highly active in HEPES or PBS, but interestingly was fastest in Tris buffer (Figure 3C). EDTA had minimal effect on SpyTag003/SpyCatcher003, while the detergents Triton X-100 or Tween 20 slightly increased the reaction rate (Figure 3C). We showed that DogTag/DogCatcher was also efficient with each partner in the nanomolar range (Figure S5).

DogTag inserted within a loop retained good DogCatcher reactivity

The Tag/Catcher approach has been used on hundreds of proteins, with the vast majority inserting the Tag at a flexible terminus of the protein of interest (Keeble and Howarth, 2019, 2020). Given that DogTag is expected to form a β hairpin to reconstitute the domain 4 structure (Figure 1A), we hypothesized that constraining DogTag at a structured internal site of a protein would allow efficient isopeptide bond formation. Therefore, we assayed DogTag inserted in an α helix in the 42 kDa HaloTag7 protein between residues 139 and 140 (Figure 4A) (Buldun et al., 2018). Comparison with reaction of a non-constrained DogTag (fused N-terminally to the MBP domain) revealed that DogTag demonstrated similar reactivity in these different environments (Figure 4B).

We also wanted to test the ability of the DogTag/DogCatcher reaction to go to completion. With 2-fold excess of DogCatcher, 98% of HaloTag7SS-DogTag reacted (Figure 4C). Conversely, with 2-fold excess of HaloTag7SS-DogTag, 98% of DogCatcher reacted (Figure 4C). We aimed to compare the reactivity of this internal DogTag with DogCatcher against internal SpyTag003 reactivity with SpyCatcher003. However, we were not able to obtain soluble expression of the construct with SpyTag003 in place of DogTag. Instead, we tried other protein scaffolds.

DogTag was superior to SpyTag003 for Catcher reactivity within superfolder GFP

The insertion of a Tag, such as SpyTag003 or DogTag, into the loop within the protein should ideally allow both high reactivity with the Catcher protein, as well as retaining the function of the host protein. In the first case, we cloned DogTag or SpyTag003 flanked on each side by G_5S linkers into loops within superfolder GFP (sfGFP) (Figure 5A), a β barrel protein previously shown permissible for loop insertions (Pavoor et al., 2009). We were pleased to find that all the variants of sfGFP were solubly expressed (with DogTag or SpyTag003 and loops A, B, or C).

We found a major difference in reactivity with the Catchers. For reaction of DogTag within loop A with DogCatcher (Figure 5B), the second-order rate constant was $1.0 \pm 0.08 \times 10^3\ M^{-1}\ s^{-1}$ (mean ± 1 SD, n = 3), which is comparable with the rate for a terminal DogTag fusion (Figure S3B). In contrast, the second-order rate constant for SpyCatcher003 reaction with SpyTag003 in the same loop of sfGFP is $87 \pm 8\ M^{-1}\ s^{-1}$ (mean ± 1 SD, n = 3), 6,000-fold slower than for SpyTag003 as a terminal fusion ($5.5 \pm 0.6 \times 10^5\ M^{-1}\ s^{-1}$) (Keeble et al., 2019).

All the loop insertion variants of sfGFP showed comparable absorption intensity and spectrum to unfused wild-type (WT) sfGFP (Figure 5C). Similarly, there was minimal change to the intensity or spectrum of fluorescence emission for any of the variants (Figure 5D). Therefore, insertion of DogTag or SpyTag003 was well tolerated for retention of fluorescent protein function.

DogTag could be inserted into loops within an enzyme while maintaining catalytic activity

sfGFP is a rigid thermostable β barrel protein, so we wanted to test an enzyme that must maintain flexibility for efficient function. The Tag/Catcher reaction has been used for scaffolding of multi-enzyme complexes and creation of catalytic hydrogels (Bitterwolf

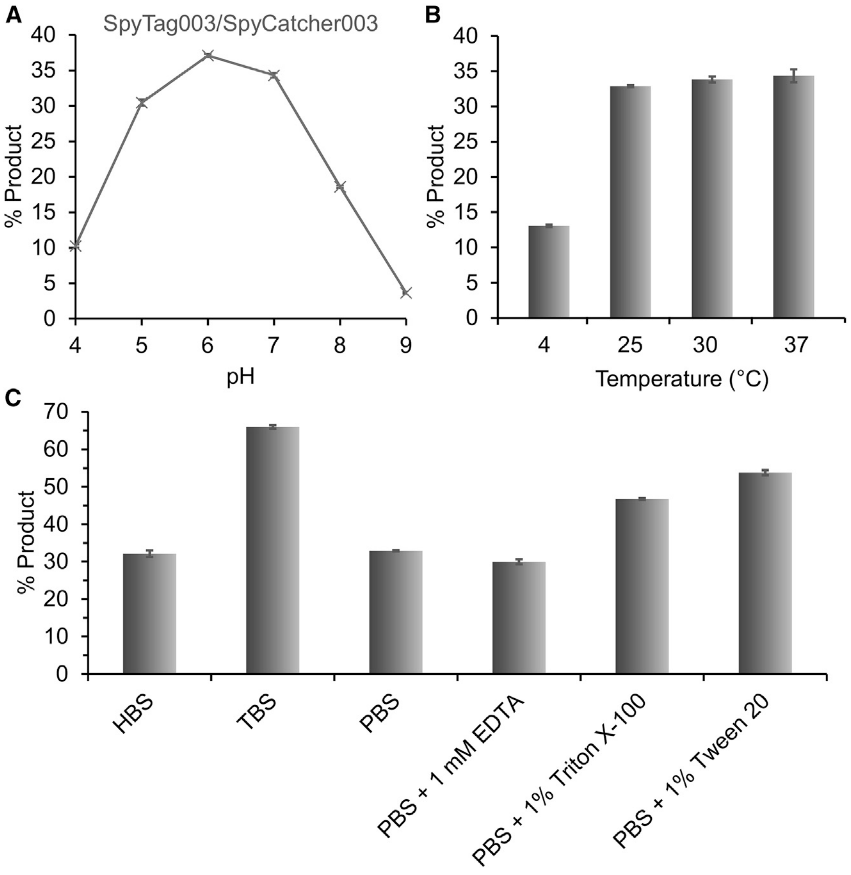

Figure 3. Condition dependence of SpyTag003/SpyCatcher003 reactivity

(A) pH dependence: 1 μM SpyTag003-MBP and 1 μM SpyCatcher003 were reacted for 15 s at 25°C in SPG buffer at the indicated pH.

(B) Temperature dependence: 100 nM SpyTag003-MBP was reacted with 100 nM SpyCatcher003-sfGFP for 2 min, pH 7.4, at the indicated temperature.

(C) Buffer dependence: 100 nM SpyTag003-MBP was reacted with 100 nM SpyCatcher003-sfGFP for 2 min in the indicated buffer, pH 7.5, at 25°C. HBS, HEPES-buffered saline; TBS, Tris-buffered saline. Mean ± 1 SD, n = 3; some error bars are too small to be visible.

et al., 2019; Liu et al., 2019). The isovaleraldehyde reductase Gre2p was used with SpyTag/SpyCatcher in this application (Bitterwolf et al., 2019) and has a mixed β-α-β Rossmann fold. We chose three loops within Gre2p away from the active site (Bitterwolf et al., 2019) to insert DogTag or SpyTag003 flanked by G_5S linkers (Figure 6A). All the insertions of SpyTag003 or DogTag allowed soluble enzyme expression. Reduction of isovaleraldehyde to isoamyl alcohol by Gre2p is nicotinamide adenine dinucleotide phosphate (NADPH) dependent (Figure 6B). We used the absorbance change upon NADPH oxidation into $NADP^+$ to follow the reaction of WT or loop-inserted Gre2p variants. With SpyTag003 or DogTag in each loop, the isovaleraldehyde reductase activity was successfully maintained within 2-fold of WT Gre2p (Figures 6C and 6D, Table S1).

For the Gre2p loop B, the second-order rate constant for the reaction of DogTag with DogCatcher was 850 ± 12 M^{-1} s^{-1}. The reaction here was much slower for SpyTag003 with SpyCatcher003 (156 ± 14 M^{-1} s^{-1}; mean ± 1 SD, n = 3, Figure 6E).

DogTag/DogCatcher orthogonality testing

SnoopTagJr/SnoopCatcher (Figure S1) is orthogonal to the SpyTag/SpyCatcher family of Tag/Catchers (Veggiani et al., 2016). We tested for cross-reactivity of DogTag/DogCatcher with SnoopTagJr/SnoopCatcher or SpyTag003/SpyCatcher003. DogTag only reacted with DogCatcher (Figure S6A), even after 24 h at high protein concentrations. DogCatcher only reacted with DogTag-containing Tag/Catcher constructs (Figure S6B). Consequently, DogCatcher did not react with SpyTag003, SpyCatcher003, or SnoopTagJr. In contrast, DogCatcher reacted to completion with HaloTag7SS-DogTag or SnoopCatcher (Figure S6B). DogCatcher reacts with SnoopCatcher because SnoopCatcher contains a sequence like DogTag at its C terminus (with DogCatcher likewise containing a sequence like SnoopTag at its N terminus, Figure S2).

DogCatcher reacted specifically with an ion channel at the mammalian cell surface

Various cell-surface proteins lack N or C termini accessible at the plasma membrane (Oberai et al., 2006). Therefore, covalent labeling with exogenous probes could be facilitated by loop-mediated ligation. Transient receptor potential canonical 5 (TRPC5) is an ion channel permeable to Na^+ and Ca^{2+} and involved in various conditions, including anxiety, kidney disease, and cardiovascular and metabolic disease (Minard et al., 2018; Wang et al., 2020). Both termini of TRPC5 are on the cytosolic side of the membrane and so we genetically inserted DogTag into the second extracellular loop between residues 460 and 461, at a site distant from the pore (Figure 7A) (Wright et al., 2020). The bright and rapidly maturing yellow fluorescent protein SYFP2 was fused to the C terminus, which allows imaging of the distribution of total TRPC5 but does not highlight the active surface pool (Bauer et al., 2020; Minard et al., 2019). To test the functionality of the DogTag insertion, we performed intracellular calcium measurements in transiently transfected HEK293 cells, stimulating TRPC5 opening with the sesquiterpinoid activator (–)-englerin A (Akbulut et al., 2015). We found that the DogTag fusion formed functional channels with efficient agonist response (Figure 7B).

We then tested the efficacy of DogCatcher recognition at the cell surface, adding biotin-DogCatcher-MBP to COS-7 cells expressing TRPC5-DogTag-SYFP2. We blotted whole-cell lysate with streptavidin-HRP, after GFP-Trap pull-down of the SYFP2 fusion. There was rapid reaction of DogCatcher with TRPC5-DogTag-SYFP2, detectable after only 1 min incubation, with minimal signal on the negative control cells lacking DogTag fusion (Figure 7C). We further tested the functionality of TRPC5 in HEK293 cells after labeling with biotin-DogCatcher-MBP. DogCatcher labeling had

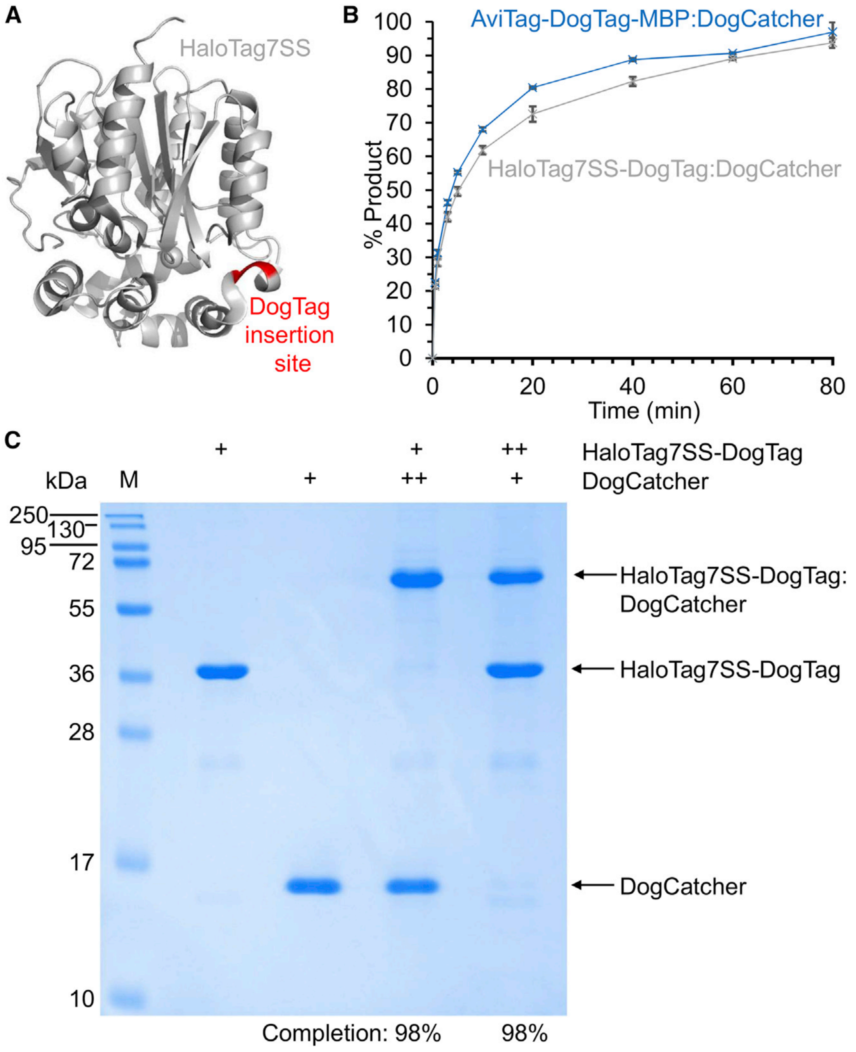

Figure 4. DogTag/DogCatcher reacted close to completion when DogTag was internal

(A) Site of DogTag insertion in red in HaloTag7 (gray, PDB: 5Y2Y).

(B) DogCatcher reaction rate with the internal DogTag in HaloTag7SS (gray trace) was similar to the unconstrained DogTag in AviTag-DogTag-MBP (blue trace). Each protein was at 5 μM in PBS, pH 7.5, at 25°C. Mean ± 1 SD, n = 3; some error bars are too small to be visible.

(C) Testing DogTag/DogCatcher reaction to completion. DogCatcher was incubated with HaloTag7SS-DogTag in PBS, pH 7.5, for 200 min at 25°C, before SDS-PAGE with Coomassie staining. +, 10 μM; ++, 20 μM; M, molecular weight markers. 98% loss was seen for HaloTag7SS-DogTag in the presence of excess DogCatcher, based on densitometry, or for DogCatcher in the presence of excess HaloTag7SS-DogTag.

no effect on TRPC5-mediated calcium influx into these cells stimulated by (−)-englerin A (Figure 7D). To visualize the surface-exposed TRPC5 pool, we first introduced a unique cysteine at the N terminus of DogCatcher and coupled maleimide-Alexa Fluor 647, to give DogCatcher-647. DogCatcher-647 allowed selective staining of TRPC5-DogTag-SYFP2 in COS-7 cells, compared with the controls lacking DogTag, with receptor visualization by confocal fluorescence microscopy. We saw DogCatcher staining as early as 1 min after addition, with optimal staining at 10 min (Figure 7E). Overall, DogTag/DogCatcher allowed rapid and selective covalent labeling of an ion channel at the surface of different mammalian cell types.

DISCUSSION

In this work we have established the DogTag/DogCatcher pair for efficient covalent protein-protein reaction in diverse protein loops. DogTag/DogCatcher showed a number of features that make the system easy to apply. Both partners are genetically encodable from the regular 20 amino acids, with reaction tolerant to a range of conditions (4°C–37°C, pH 6–8, detergents, or different buffers). Reaction can proceed to ∼98% conversion without detectable side products and leaves an amide bond that is anticipated to have high stability. Neither DogTag nor DogCatcher contains cysteine, so coupling can be performed on proteins requiring reducing or oxidizing conditions.

We demonstrated efficient reaction for DogTag at the terminus of a protein, or with DogTag inserted internally in predominantly α-helical, α+β or predominantly β sheet proteins. We also showed maintenance of good fluorescence characteristics when inserted in different loops of sfGFP and good catalytic activity in different loops of Gre2p. In the case of HaloTag, DogTag was inserted within a secondary structure element.

For the generation of Tag/Catcher pairs, the work here fits with the literature that initial designs of split proteins often successfully reconstitute (Shekhawat and Ghosh, 2011). However, it is a considerable challenge to obtain Tag/Catcher pairs with rapid and high-yielding reaction. The majority of Tag/Catcher pairs in the literature require high micromolar concentration and days for substantial coupling (Bonnet et al., 2017; Proschel et al., 2017; Veggiani et al., 2016; Wu et al., 2018; Zakeri and Howarth, 2010; Zhang et al., 2018). Therefore, substantial protein engineering effort is required to achieve rapid spontaneous intermolecular isopeptide bond formation. SpyTag003/SpyCatcher003 has received such devoted optimization, enabling reaction at close to the diffusion limit with SpyTag003 at protein termini (Keeble et al., 2019). It was encouraging that protein function was retained with SpyTag003 inserted in these constrained loop environments. However, it is striking how reaction may be decreased with SpyCatcher003 by three orders of magnitude when SpyTag003 is in different internal loops. Therefore, the application here of DogTag, based on a β hairpin, with DogCatcher led to a preferred pairing for reaction with various loops. This difference in target conformation is consistent with results on epitope tags. The

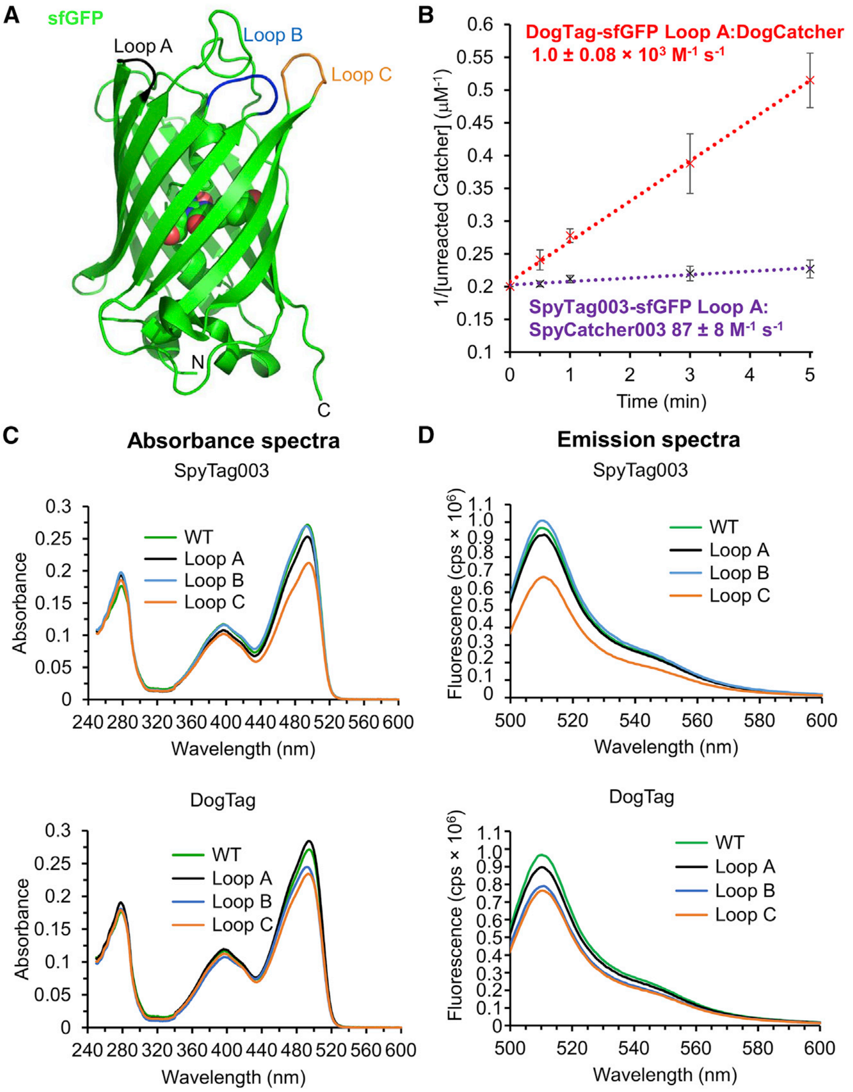

Figure 5. DogTag functioned well within the β barrel domain of sfGFP and reacted faster than SpyTag003

(A) Structure of sfGFP (PDB: 2B3P) showing the three loops chosen for tag insertion.

(B) Second-order reaction plot comparing the reaction speed of DogCatcher with DogTag in sfGFP loop A (red trace), relative to SpyCatcher003 reaction with SpyTag003 (purple trace) in PBS, pH 7.5, at 25°C. Mean ± 1 SD, n = 3. Some error bars are too small to be visible.

(C) Comparison of the absorbance spectra of sfGFP (WT) or variants with SpyTag003 or DogTag at the indicated loop.

(D) Comparison of the fluorescence emission of sfGFP (WT) or variants with SpyTag003 or DogTag at the indicated loop upon excitation at 488 nm. cps, counts per second.

MAP tag forms a hairpin in its complex with an antibody: proteins with a MAP tag inserted at internal loops displayed good retention of activity of the fused protein and efficient antibody detection (Fujii et al., 2016; Wakasa et al., 2020). We found that the rate of DogTag/DogCatcher reaction was comparable at a terminal site or a loop site. This similarity may reflect a balance between increased accessibility at the terminus, versus preformation of DogTag's reactive conformation increasing the reactivity at a loop site. It is beyond our scope here to perform biophysical analysis of the conformational flexibility of these DogTag insertions, but further fusion constructs may illuminate this question. Our optimization led to a 250-fold increase in reaction rate. Adding to the challenge, we need to take a different strategy for the optimization of DogCatcher compared with optimizing SnoopLigase peptide-peptide ligation (Buldun et al., 2018), suggesting that these two proteins faced different challenges for efficient reconstitution. The slow reaction of SnoopLigase (~48 h to reach completion) (Buldun et al., 2018) limits its application, especially in cellular systems. We achieved increase in DogCatcher's solubility and reaction rate based upon proline-based reduction in loop flexibility, increase in surface negative charge to enhance solubility, and phage display evolution. As is often found with directed evolution (Bloom and Arnold, 2009), phage selection identified mutations in DogCatcher distant from the site of direct interaction with DogTag. The increased reaction speed of DogCatcher following this optimization may be important for time resolution in cell biology and coupling to low abundance partners (Keeble et al., 2019). DogTag/DogCatcher reacts faster and at lower protein concentrations than the related SnoopTag/SnoopCatcher pair, which also requires stabilizing chemicals, such as 1.5 M trimethylamine N-oxide for optimal reaction (Veggiani et al., 2016). Therefore, DogTag/DogCatcher represents a highly loop-friendly technology. Disadvantages of DogTag/DogCatcher include the size (23 and 104 residues) and the anticipated immunogenicity based on the bacterial origin (Rahikainen et al., 2020). However, fewer people had pre-existing antibodies to SnoopTag/SnoopCatcher (created from an alternative splitting of RrgA domain 4) than SpyTag/SpyCatcher (Rahikainen et al., 2020).

Numerous proteins are not amenable to fusion at termini, including those with termini key for function (Huh et al., 2003) or buried at interprotein interfaces (e.g., Qβ virus-like particles) (Golmohammadi et al., 1996). Many membrane proteins have both termini on the intracellular side of the plasma membrane, including most ABC transporters, the major facilitator superfamily, bacterial outer membrane porins, and tetraspanins (Oberai et al., 2006). Here, we have shown that DogTag can be inserted in an exposed loop of the ion channel protein TRPC5 and can react with DogCatcher without apparent disruption of function, based on agonist-induced activation of calcium influx. DogCatcher allowed rapid and specific labeling of TRPC5 at the mammalian cell surface, based on western blotting and fluorescence microscopy. It has proved challenging to generate antibodies to the extracellular region of TRPC5, like many other

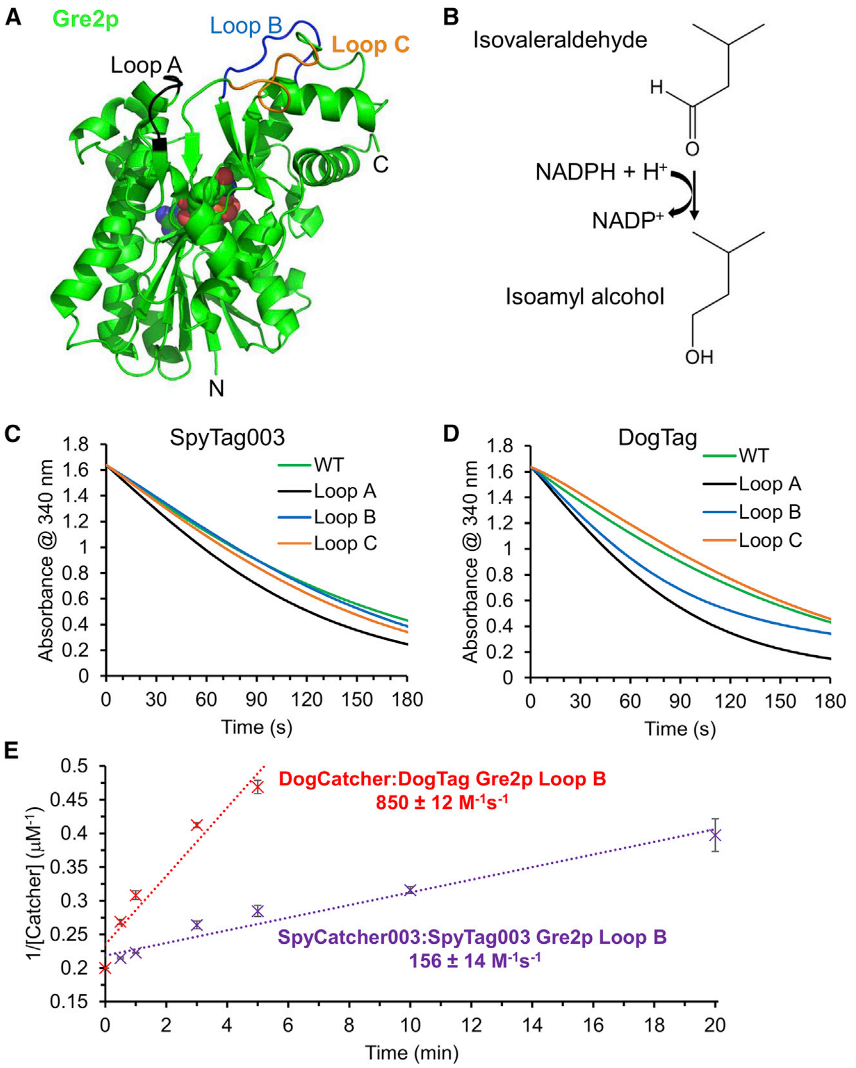

Figure 6. Tag reactivity and enzyme activity after loop insertion

(A) Structure of Gre2p showing the three loops chosen for tag insertion (PDB: 4PVD). NADPH is shown as spheres.

(B) Schematic of the reaction catalyzed by Gre2p.

(C) SpyTag003 loop insertion had little effect on enzyme activity. Comparison of isovaleraldehyde reductase activity of the Gre2p variants, assayed by the decrease in absorbance at 340 nm as NADPH is converted into $NADP^+$. Data represent the mean of three biological replicates.

(D) DogTag loop insertion had little effect on enzyme activity, assayed as in (C). Data represent the mean of three biological replicates.

(E) DogTag/DogCatcher (red trace) reacted faster than SpyTag003/SpyCatcher003 (purple trace) in loop B of Gre2p. Second-order reaction plot in PBS, pH 7.5, at 25°C. Mean ± 1 SD, n = 3. Some error bars are too small to be visible.

proteins with short exposed surface loops, and such antibodies will have limited stability in their recognition, compared with the covalent DogCatcher reaction. In contrast to covalent modification with DogCatcher, antibodies generated against TRPC5's longer third extracellular loop inhibit TRPC5 function (Xu et al., 2005). Many other ion channels also do not have surface-exposed termini, including voltage-gated potassium, sodium, and calcium channels (Anderson and Greenberg, 2001), so this route to covalent derivatization could have broad application for ion channel analysis. Even when termini are a possible fusion site, loop fusion may still be preferred to control protein orientation, such as in diagnostics, multi-enzyme complexes, or vaccine conjugates (Akiba et al., 2020; Brune and Howarth, 2018; Sweetlove and Fernie, 2018). Therefore, DogTag/DogCatcher should be a significant addition to the toolbox for synthetic biology.

SIGNIFICANCE

Peptide tags enable powerful generic approaches for protein detection and modification. The majority of peptide tags have been validated for fusion to the N terminus or C terminus of protein targets. In this work we have focused on reaction at internal sites in proteins, which are often more challenging. We previously established spontaneous isopeptide bond formation to allow rapid covalent reaction between SpyTag003 and its protein partner SpyCatcher003. Here, we have genetically split the *Streptococcus pneumoniae* adhesin RrgA to give an orthogonal Tag/Catcher pair, DogTag and DogCatcher. Through structure-based design and phage display evolution, we were able to increase the reaction rate by 250-fold and establish high solubility of DogCatcher. We found that DogTag/DogCatcher reacted efficiently in a broad range of conditions and was tolerant of a range of buffers, temperatures, and pH values. To test the performance of tags in loops, we inserted DogTag or SpyTag003 at various sites in a fluorescent protein or an enzyme. Fluorescence brightness or enzyme activity was little changed by these fusions. SpyTag003/SpyCatcher003 gave much faster reaction than DogTag/DogCatcher when the tag was fused to a protein terminus. However, in loops of a fluorescent protein or enzyme, we found that DogTag/DogCatcher reacted a lot faster than SpyTag003/SpyCatcher003. We then evaluated reactivity at the surface of mammalian cells. In common with a range of membrane proteins, the ion channel TRPC5 has no termini exposed to the outside of the cell. The fusion of DogTag in a TRPC5 extracellular loop led to normal calcium flux and we detected specific covalent labeling on cells in 1 min using DogCatcher. DogTag and DogCatcher are genetically encodable, using only the normal amino acids, and so their efficient reaction opens up a simple and scalable route to irreversible modification at internal sites of proteins.

CellPress
OPEN ACCESS

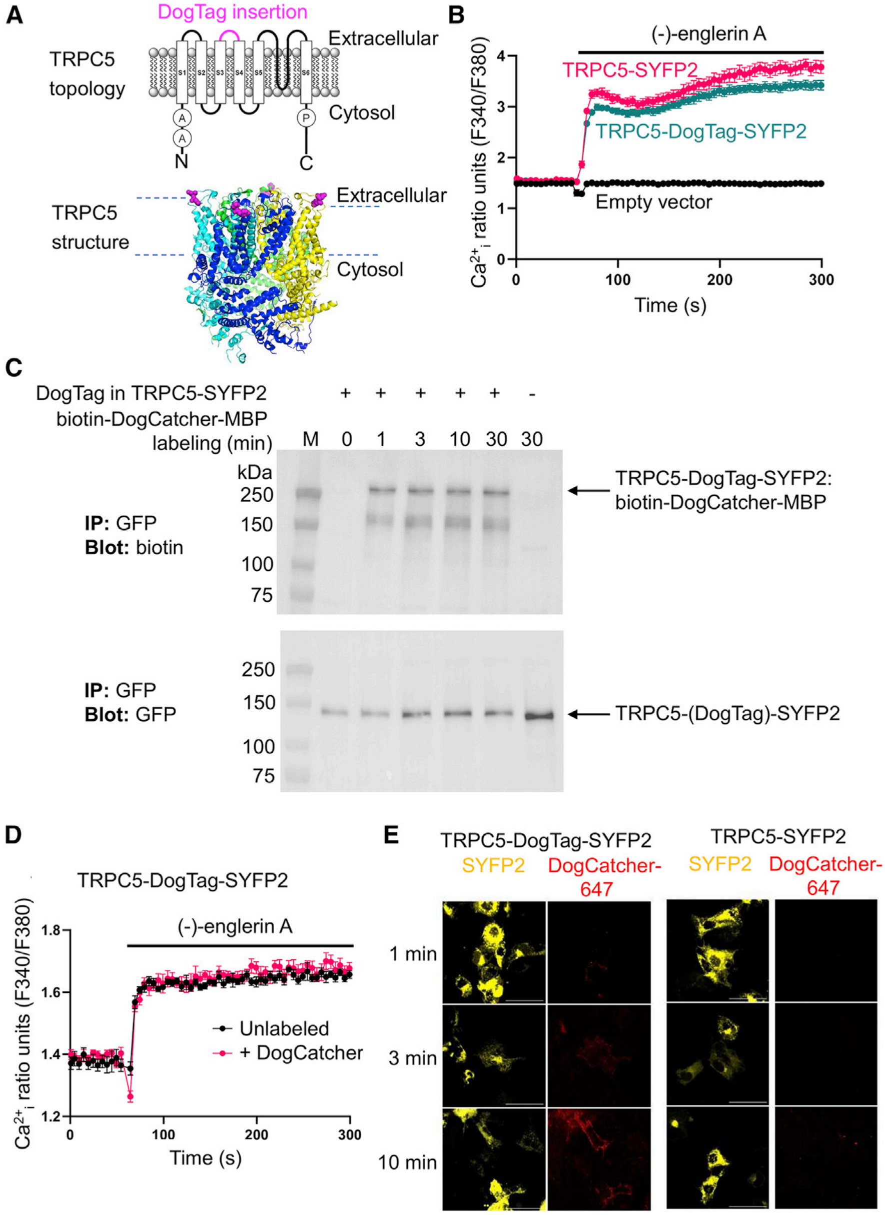

Figure 7. Specific targeting of an ion channel using DogTag/DogCatcher

(A) Schematic of TRPC5, with the insertion site (pink) of DogTag in the second extracellular loop marked on a topology diagram and a crystal structure (PDB: 6YSN, each chain of the tetramer in a different color). A, ankyrin repeat domain; P, TRP domain.

(B) DogTag insertion had minimal effect on ion channel opening. Representative intracellular calcium measurements ($Ca^{2+}{}_i$) from one 96-well plate (mean ± 1 SE, n = 4) showing activation of TRPC5-SYFP2 (red trace) or TRPC5-DogTag-SYFP2 (teal trace) in HEK 293 cells by 30 nM (−)-englerin A (present during the period marked with a horizontal line). No calcium response was induced by (−)-englerin A in empty vector-transfected cells (black trace).

(C) Rapid labeling by DogCatcher at the cell surface. COS-7 cells expressing TRPC5-DogTag-SYFP2 or TRPC5-SYFP2 control were incubated with 5 μM biotin-DogCatcher-MBP for the indicated time at 25°C. Cell lysates were immunoprecipitated with GFP-Trap before blotting for either biotin (top panel) or fluorescent protein (bottom panel).

(D) DogCatcher reaction had minimal effect on ion channel opening. Representative intracellular calcium measurements ($Ca^{2+}{}_i$) from one 96-well plate (mean ± 1 SE, n = 6) showing activation of TRPC5-DogTag-SYFP2 in HEK293 cells by 10 nM (−)-englerin A (present during the period marked with a horizontal line), with (red trace) or without (black trace) 30 min pre-treatment with 5 μM biotin-DogCatcher-MBP.

(E) DogCatcher labeled specifically at the cell surface: 5 μM DogCatcher-647 was incubated for varying times at 25°C with live COS-7 cells expressing TRPC5-DogTag-SYFP2 or TRPC5-SYFP2, before fixation and confocal microscopy. Images represent confocal slices, with SYFP2 in yellow and DogCatcher-647 in red. Scale bar, 50 μm.

STAR★METHODS

Detailed methods are provided in the online version of this paper and include the following:

- KEY RESOURCES TABLE
- RESOURCE AVAILABILITY
  - Lead contact
  - Material availability
  - Data and code availability
- EXPERIMENTAL MODEL AND SUBJECT DETAILS
- METHOD DETAILS
  - Bacterial strain
  - Cell lines
  - Cloning of constructs
  - Protein expression and purification
  - R2CatcherB WT phage production
  - Phage library generation
  - Phage selections
  - Isopeptide bond formation assays
  - Protein yield and solubility determination
  - DogCatcher dye labeling
  - Spectroscopic measurements of sfGFP
  - Mass spectrometry
  - Gre2p activity assay
  - Intracellular calcium measurement
  - GFP-trap and Western blot
  - Fluorescence microscopy
  - Structure visualization
- QUANTIFICATION AND STATISTICAL ANALYSIS
  - Statistical analysis

SUPPLEMENTAL INFORMATION

Supplemental information can be found online at https://doi.org/10.1016/j.chembiol.2021.07.005.

ACKNOWLEDGMENTS

Funding was provided by the Engineering and Physical Sciences Research Council (EPSRC EP/N023226/1) (to M.P.F., V.K.Y., and M.H.) and Biotechnology and Biological Sciences Research Council (BBSRC BB/S007369/1) (to A.H.K. and M.H.). C.C.B., E.C.-B., and R.S.B. were funded by the British Heart Foundation (PG/19/2/34084 and SI/14/1/30718) and the Wellcome Trust (10044/Z/15/Z). We thank Dr. David Staunton of the University of Oxford Department of Biochemistry Biophysical Suite for assistance with biophysical analysis and Dr. David Wright of Leeds Institute for Cardiovascular and Metabolic Medicine for assistance with TRPC5 construct design. We acknowledge Dr. Anthony Tumber of the University of Oxford Department of Chemistry for assistance with MS, supported by the BBSRC (BB/R000344/1).

AUTHOR CONTRIBUTIONS

M.P.F., V.K.Y., and J.H. performed engineering and selection to generate DogCatcher. A.H.K. carried out *in vitro* testing of DogTag/DogCatcher and SpyTag003/SpyCatcher003 behavior. C.C.B. and E.C.-B. generated TRPC5-DogTag-SYFP2 and performed cellular analysis on TRPC5. R.S.B. supervised the TRPC5 research. M.H. conceived and supervised the research. A.H.K. and M.H. wrote the manuscript. All authors approved the manuscript.

DECLARATION OF INTERESTS

M.H. and J.H. are authors on a patent covering RrgA splitting (UK Intellectual Property Office 1509782.7). M.H. is an author on a patent covering DogTag and SnoopLigase (UK Intellectual Property Office 1705750.6). M.H. and A.H.K. are authors on a patent application for SpyTag003/SpyCatcher003 (UK Intellectual Property Office, 1903479.2). M.H., A.H.K., M.P.F., and V.K.Y. are authors on a patent application related to DogCatcher (UK Intellectual Property Office 2104999.4.). M.H. is a SpyBiotech co-founder, shareholder, and consultant. All other authors have no conflicts of interest.

Received: April 12, 2021
Revised: June 9, 2021
Accepted: July 2, 2021
Published: July 28, 2021

REFERENCES

Akbulut, Y., Gaunt, H.J., Muraki, K., Ludlow, M.J., Amer, M.S., Bruns, A., Vasudev, N.S., Radtke, L., Willot, M., Hahn, S., et al. (2015). (–)-Englerin A is a potent and selective activator of TRPC4 and TRPC5 calcium channels. Angew. Chem. *54*, 3787–3791.

Akiba, H., Takayanagi, K., Kusano-Arai, O., Iwanari, H., Hamakubo, T., and Tsumoto, K. (2020). Generation of biparatopic antibody through two-step targeting of fragment antibodies on antigen using SpyTag and SpyCatcher. Biotechnol. Rep. *25*, e00418.

Anderson, P.A., and Greenberg, R.M. (2001). Phylogeny of ion channels: clues to structure and function. Comp. Biochem. Physiol. B Biochem. Mol. Biol. *129*, 17–28.

Bae, Y., Lee, S.K., Chae, Y.C., Park, C.Y., and Kang, S. (2021). Accessibility-dependent topology studies of membrane proteins using a SpyTag/SpyCatcher protein-ligation system. Int. J. Biol. Macromol. *175*, 171–178.

Banerjee, A., and Howarth, M. (2018). Nanoteamwork: covalent protein assembly beyond duets towards protein ensembles and orchestras. Curr. Opin. Biotechnol. *51*, 16–23.

Baneyx, F., and Mujacic, M. (2004). Recombinant protein folding and misfolding in *Escherichia coli*. Nat. Biotechnol. *22*, 1399–1408.

Bauer, C.C., Minard, A., Pickles, I.B., Simmons, K.J., Chuntharpursat-Bon, E., Burnham, M.P., Kapur, N., Beech, D.J., Muench, S.P., Wright, M.H., et al. (2020). Xanthine-based photoaffinity probes allow assessment of ligand engagement by TRPC5 channels. RSC Chem. Biol. *1*, 436–448.

Beekwilder, J., Rakonjac, J., Jongsma, M., and Bosch, D. (1999). A phagemid vector using the *E. coli* phage shock promoter facilitates phage display of toxic proteins. Gene *228*, 23–31.

Bitterwolf, P., Gallus, S., Peschke, T., Mittmann, E., Oelschlaeger, C., Willenbacher, N., Rabe, K.S., and Niemeyer, C.M. (2019). Valency engineering of monomeric enzymes for self-assembling biocatalytic hydrogels. Chem. Sci. *10*, 9752–9757.

Bloom, J.D., and Arnold, F.H. (2009). In the light of directed evolution: pathways of adaptive protein evolution. Proc. Natl. Acad. Sci. U S A *106*, 9995–10000.

Bonnet, J., Cartannaz, J., Tourcier, G., Contreras-Martel, C., Kleman, J.P., Morlot, C., Vernet, T., and Di Guilmi, A.M. (2017). Autocatalytic association of proteins by covalent bond formation: a Bio Molecular Welding toolbox derived from a bacterial adhesin. Scientific Rep. *7*, 43564.

Brune, K.D., and Howarth, M. (2018). New routes and opportunities for modular construction of particulate vaccines: stick, click, and glue. Front. Immunol. *9*, 1432.

Buldun, C.M., Jean, J.X., Bedford, M.R., and Howarth, M. (2018). SnoopLigase catalyzes peptide-peptide locking and enables solid-phase conjugate isolation. J. Am. Chem. Soc. *140*, 3008–3018.

Cabrita, L.D., Gilis, D., Robertson, A.L., Dehouck, Y., Rooman, M., and Bottomley, S.P. (2007). Enhancing the stability and solubility of TEV protease using in silico design. Protein Sci. *16*, 2360–2367.

Correnti, C.E., Gewe, M.M., Mehlin, C., Bandaranayake, A.D., Johnsen, W.A., Rupert, P.B., Brusniak, M.Y., Clarke, M., Burke, S.E., De Van Der Schueren, W., et al. (2018). Screening, large-scale production and structure-based classification of cystine-dense peptides. Nat. Struct. Mol. Biol. *25*, 270–278.

Fairhead, M., and Howarth, M. (2015). Site-specific biotinylation of purified proteins using BirA. Methods Mol. Biol. *1266*, 171–184.

Fu, H., Grimsley, G.R., Razvi, A., Scholtz, J.M., and Pace, C.N. (2009). Increasing protein stability by improving beta-turns. Proteins *77*, 491–498.

Fujii, Y., Matsunaga, Y., Arimori, T., Kitago, Y., Ogasawara, S., Kaneko, M.K., Kato, Y., and Takagi, J. (2016). Tailored placement of a turn-forming PA tag into the structured domain of a protein to probe its conformational state. J. Cell Sci. *129*, 1512–1522.

Gasteiger, E., Hoogland, C., Gattiker, A., Duvaud, S., Wilkins, M.R., Appel, R.D., and Bairoch, A. (2005). Protein identification and analysis tools on the ExPASy server. In The Proteomics Protocols Handbook, J.M. Walker, ed. (Humana Press), pp. 571–607.

Goldenzweig, A., Goldsmith, M., Hill, S.E., Gertman, O., Laurino, P., Ashani, Y., Dym, O., Unger, T., Albeck, S., Prilusky, J., et al. (2016). Automated structure- and sequence-based design of proteins for high bacterial expression and stability. Mol. Cell *63*, 337–346.

Golmohammadi, R., Fridborg, K., Bundule, M., Valegard, K., and Liljas, L. (1996). The crystal structure of bacteriophage Q beta at 3.5 A resolution. Structure *4*, 543–554.

Guo, P.C., Bao, Z.Z., Ma, X.X., Xia, Q., and Li, W.F. (2014). Structural insights into the cofactor-assisted substrate recognition of yeast methylglyoxal/isovaleraldehyde reductase Gre2. Biochim. Biophys. Acta *1844*, 1486–1492.

Hentrich, C., Kellmann, S.J., Putyrski, M., Cavada, M., Hanuschka, H., Knappik, A., and Ylera, F. (2021). Periplasmic expression of SpyTagged antibody fragments enables rapid modular antibody assembly. Cell Chem. Biol. *28*, 813–824.e6.

Hofmann, R., Akimoto, G., Wucherpfennig, T.G., Zeymer, C., and Bode, J.W. (2020). Lysine acylation using conjugating enzymes for site-specific modification and ubiquitination of recombinant proteins. Nat. Chem. *12*, 1008–1015.

Huh, W.K., Falvo, J.V., Gerke, L.C., Carroll, A.S., Howson, R.W., Weissman, J.S., and O'Shea, E.K. (2003). Global analysis of protein localization in budding yeast. Nature *425*, 686–691.

Izore, T., Contreras-Martel, C., El Mortaji, L., Manzano, C., Terrasse, R., Vernet, T., Di Guilmi, A.M., and Dessen, A. (2010). Structural basis of host cell recognition by the pilus adhesin from *Streptococcus pneumoniae*. Structure *18*, 106–115.

Jacob, E., and Unger, R. (2007). A tale of two tails: why are terminal residues of proteins exposed? Bioinformatics *23*, e225–230.

Jbara, M., Sun, H., Kamnesky, G., and Brik, A. (2018). Chemical chromatin ubiquitylation. Curr. Opin. Chem. Biol. *45*, 18–26.

Kang, M.G., Lee, H., Kim, B.H., Dunbayev, Y., Seo, J.K., Lee, C., and Rhee, H.W. (2017). Structure-guided synthesis of a protein-based fluorescent sensor for alkyl halides. Chem. Commun. *53*, 9226–9229.

Kapust, R.B., Tozser, J., Fox, J.D., Anderson, D.E., Cherry, S., Copeland, T.D., and Waugh, D.S. (2001). Tobacco etch virus protease: mechanism of autolysis and rational design of stable mutants with wild-type catalytic proficiency. Protein Eng. *14*, 993–1000.

Keeble, A.H., Banerjee, A., Ferla, M.P., Reddington, S.C., Anuar, I., and Howarth, M. (2017). Evolving accelerated amidation by SpyTag/SpyCatcher to analyze membrane dynamics. Angew. Chem. *56*, 16521–16525.

Keeble, A.H., and Howarth, M. (2019). Insider information on successful covalent protein coupling with help from SpyBank. Methods Enzymol. *617*, 443–461.

Keeble, A.H., and Howarth, M. (2020). Power to the protein: enhancing and combining activities using the Spy toolbox. Chem. Sci. *11*, 7281–7291.

Keeble, A.H., Turkki, P., Stokes, S., Khairil Anuar, I.N.A., Rahikainen, R., Hytonen, V.P., and Howarth, M. (2019). Approaching infinite affinity through engineering of peptide-protein interaction. Proc. Natl. Acad. Sci. U S A *116*, 26523–26533.

Knott, B.C., Erickson, E., Allen, M.D., Gado, J.E., Graham, R., Kearns, F.L., Pardo, I., Topuzlu, E., Anderson, J.J., Austin, H.P., et al. (2020). Characterization and engineering of a two-enzyme system for plastics depolymerization. Proc. Natl. Acad. Sci. U S A *117*, 25476–25485.

Leaver-Fay, A., Tyka, M., Lewis, S.M., Lange, O.F., Thompson, J., Jacak, R., Kaufman, K., Renfrew, P.D., Smith, C.A., Sheffler, W., et al. (2011). ROSETTA3: an object-oriented software suite for the simulation and design of macromolecules. Methods Enzymol. *487*, 545–574.

Li, L., Fierer, J.O., Rapoport, T.A., and Howarth, M. (2014). Structural analysis and optimization of the covalent association between SpyCatcher and a peptide Tag. J. Mol. Biol. *426*, 309–317.

Liu, Z., Cao, S., Liu, M., Kang, W., and Xia, J. (2019). Self-assembled multienzyme nanostructures on synthetic protein scaffolds. ACS Nano *13*, 11343–11352.

Lovell, S.C., Davis, I.W., Arendall, W.B., 3rd, de Bakker, P.I., Word, J.M., Prisant, M.G., Richardson, J.S., and Richardson, D.C. (2003). Structure validation by Calpha geometry: phi, psi and Cbeta deviation. Proteins *50*, 437–450.

Manandhar, M., Chun, E., and Romesberg, F.E. (2021). Genetic code expansion: inception, development, commercialization. J. Am. Chem. Soc. *143*, 4859–4878.

Minard, A., Bauer, C.C., Chuntharpursat-Bon, E., Pickles, I.B., Wright, D.J., Ludlow, M.J., Burnham, M.P., Warriner, S.L., Beech, D.J., Muraki, K., et al. (2019). Potent, selective, and subunit-dependent activation of TRPC5 channels by a xanthine derivative. Br. J. Pharmacol. *176*, 3924–3938.

Minard, A., Bauer, C.C., Wright, D.J., Rubaiy, H.N., Muraki, K., Beech, D.J., and Bon, R.S. (2018). Remarkable progress with small-molecule modulation of TRPC1/4/5 channels: implications for understanding the channels in health and disease. Cells *7*, 52.

Morris, C., Glennie, S.J., Lam, H.S., Baum, H.E., Kandage, D., Williams, N.A., Morgan, D.J., Woolfson, D.N., and Davidson, A.D. (2019). A modular vaccine platform combining self-assembled peptide cages and immunogenic peptides. Adv. Funct. Mater. *29*, 1807357.

Oberai, A., Ihm, Y., Kim, S., and Bowie, J.U. (2006). A limited universe of membrane protein families and folds. Protein Sci. *15*, 1723–1734.

Oesterle, S., Roberts, T.M., Widmer, L.A., Mustafa, H., Panke, S., and Billerbeck, S. (2017). Sequence-based prediction of permissive stretches for internal protein tagging and knockdown. BMC Biol. *15*, 100.

Oke, M., Carter, L.G., Johnson, K.A., Liu, H., Mcmahon, S.A., Yan, X., Kerou, M., Weikart, N.D., Kadi, N., Sheikh, M.A., et al. (2010). The Scottish Structural Proteomics Facility: targets, methods and outputs. J. Struct. Funct. Genomics *11*, 167–180.

Pavoor, T.V., Cho, Y.K., and Shusta, E.V. (2009). Development of GFP-based biosensors possessing the binding properties of antibodies. Proc. Natl. Acad. Sci. U S A *106*, 11895–11900.

Pedelacq, J.D., Cabantous, S., Tran, T., Terwilliger, T.C., and Waldo, G.S. (2006). Engineering and characterization of a superfolder green fluorescent protein. Nat. Biotechnol. *24*, 79–88.

Proschel, M., Kraner, M.E., Horn, A.H.C., Schafer, L., Sonnewald, U., and Sticht, H. (2017). Probing the potential of CnaB-type domains for the design of Tag/Catcher systems. PLoS One *12*, e0179740.

Qu, J., Cao, S., Wei, Q., Zhang, H., Wang, R., Kang, W., Ma, T., Zhang, L., Liu, T., Wing-Ngor Au, S., et al. (2019). Synthetic multienzyme complexes, catalytic nanomachineries for cascade biosynthesis in vivo. ACS Nano *13*, 9895–9906.

Rahikainen, R., Rijal, P., Tan, T.K., Wu, H.J., Andersson, A.M.C., Barrett, J.R., Bowden, T.A., Draper, S.J., Townsend, A.R., and Howarth, M. (2020). Overcoming symmetry mismatch in vaccine nanoassembly through spontaneous amidation. Angew. Chem. Int. Edit *60*, 321–330.

Schneider, H., Deweid, L., Avrutina, O., and Kolmar, H. (2020). Recent progress in transglutaminase-mediated assembly of antibody-drug conjugates. Anal. Biochem. *595*, 113615.

Shekhawat, S.S., and Ghosh, I. (2011). Split-protein systems: beyond binary protein-protein interactions. Curr. Opin. Chem. Biol. *15*, 789–797.

Steiner, D., Forrer, P., Stumpp, M.T., and Pluckthun, A. (2006). Signal sequences directing cotranslational translocation expand the range of proteins amenable to phage display. Nat. Biotechnol. *24*, 823–831.

Sormanni, P., Aprile, F.A., and Vendruscolo, M. (2015). The CamSol method of rational design of protein mutants with enhanced solubility. J. Mol. Biol. *427*, 478–490.

Sue, C.K., McConnell, S.A., Ellis-Guardiola, K., Muroski, J.M., McAllister, R.A., Yu, J., Alvarez, A.I., Chang, C., Ogorzalek Loo, R.R., Loo, J.A., et al. (2020). Kinetics and optimization of the lysine-isopeptide bond forming sortase enzyme from *Corynebacterium diphtheriae*. Bioconjug. Chem. *31*, 1624–1634.

Sweetlove, L.J., and Fernie, A.R. (2018). The role of dynamic enzyme assemblies and substrate channelling in metabolic regulation. Nat. Commun. *9*, 2136.

Trevino, S.R., Schaefer, S., Scholtz, J.M., and Pace, C.N. (2007). Increasing protein conformational stability by optimizing beta-turn sequence. J. Mol. Biol. *373*, 211–218.

Veggiani, G., Nakamura, T., Brenner, M.D., Gayet, R.V., Yan, J., Robinson, C.V., and Howarth, M. (2016). Programmable polyproteams built using twin peptide superglues. Proc. Natl. Acad. Sci. U S A *113*, 1202–1207.

Wakasa, A., Kaneko, M.K., Kato, Y., Takagi, J., and Arimori, T. (2020). Site-specific epitope insertion into recombinant proteins using the MAP tag system. J. Biochem. *168*, 375–384.

Wang, H., Cheng, X., Tian, J., Xiao, Y., Tian, T., Xu, F., Hong, X., and Zhu, M.X. (2020). TRPC channels: structure, function, regulation and recent advances in small molecular probes. Pharmacol. Ther. *209*, 107497.

Wright, D.J., Simmons, K.J., Johnson, R.M., Beech, D.J., Muench, S.P., and Bon, R.S. (2020). Human TRPC5 structures reveal interaction of a xanthine-based TRPC1/4/5 inhibitor with a conserved lipid binding site. Commun. Biol. *3*, 704.

Wu, X.L., Liu, Y., Liu, D., Sun, F., and Zhang, W.B. (2018). An intrinsically disordered peptide-peptide stapler for highly efficient protein ligation both in vivo and in vitro. J. Am. Chem. Soc. *140*, 17474–17483.

Xu, S.Z., Zeng, F., Lei, M., Li, J., Gao, B., Xiong, C., Sivaprasadarao, A., and Beech, D.J. (2005). Generation of functional ion-channel tools by E3 targeting. Nat. Biotechnol. *23*, 1289–1293.

Zakeri, B., Fierer, J.O., Celik, E., Chittock, E.C., Schwarz-Linek, U., Moy, V.T., and Howarth, M. (2012). Peptide tag forming a rapid covalent bond to a protein, through engineering a bacterial adhesin. Proc. Natl. Acad. Sci. U S A *109*, E690–E697.

Zakeri, B., and Howarth, M. (2010). Spontaneous intermolecular amide bond formation between side chains for irreversible peptide targeting. J. Am. Chem. Soc. *132*, 4526–4527.

Zhang, X.J., Wu, X.L., Wang, X.W., Liu, D., Yang, S., and Zhang, W.B. (2018). SpyCatcher-N(TEV): a circularly permuted, disordered SpyCatcher variant for less trace ligation. Bioconjug. Chem. *29*, 1622–1629.

STAR★METHODS

KEY RESOURCES TABLE

REAGENT or RESOURCE	SOURCE	IDENTIFIER
Antibodies		
Mouse anti-GFP	Abcam	Cat# ab1218, RRID:AB_298911
Rabbit anti-Mouse IgG (H+L) Secondary Antibody, horseradish peroxidase	Thermo Fisher	Cat# A16160,RRID: AB_2534831
Bacterial and virus strains		
E. coli BL21 (DE3) RIPL	Agilent	Cat#230280
E. coli XL1-Blue	Agilent	Cat#200236
E. coli K12 ER2738	Lucigen	Cat#60522-1
R408 helper phage	Agilent	Cat#200252
E. coli NEB Turbo	New England Biolabs	C2984H
E. coli C41 (DE3)	A gift from Anthony Watts, University of Oxford	N/A
Chemicals, peptides, and recombinant proteins		
Alexa Fluor 647 C2 Maleimide	Thermo Fisher	Cat#A20347 CAS:541-59-3
Biotin-Binder Dynabeads	Thermo Fisher	Cat# 11047
Bovine serum albumin (BSA)	Merck	Cat#A9418 CAS:9048-46-8
Dimethylsulfoxide (DMSO)	Merck	Cat#D8418 CAS:67-68-5
DL-Dithiothreitol (DTT)	Merck	Cat#D9779 CAS: 3483-12-3
Dulbecco's Modified Eagle Medium	Thermo Fisher	Cat#31966047
(−)-Englerin A	PhytoLab	Cat#82530 - 10MG; CAS 1094250-15-3
Fetal Bovine Serum	Merck	Cat#F9665-500ML
Fura-2, AM, cell permeant	Thermo Fisher	Cat# F1221 CAS:108964-32-5
GFP-Trap agarose	Chromotek	Cat# gta-10
InstantBlue Protein Stain	Expedeon	Cat#ISB1L
Isopropyl-β-D-thiogalactoside (IPTG)	Fluorochem	Cat# M02726-25G CAS: 367-93-1
Isovaleraldehyde	Merck	Cat#146455 CAS: 590-86-3
jetPRIME® DNA and siRNA transfection reagent	VWR	Cat#114-07
Nicotinamide adenine dinucleotide phosphate (NADPH) tetrasodium salt	Santa Cruz Biotechnology	Cat# sc-202725 CAS:2646-71-1
β-mercaptoethanol	Merck	Cat#M6250
Paraformaldehyde, 4% in PBS	Alfa Aesar	Cat# J61899.AK
Penicillin-Streptomycin (10,000 U/mL)	Thermo Fisher	Cat#15140122
Pierce ECL	Thermo Fisher	Cat#32209
Phenylmethylsulfonyl fluoride	Thermo Fisher	Cat#36978; CAS:329-98-6
Pluronic acid (Pluronic® F-127)	Merck	Cat#P2443
Polyethylene glycol 8000	Fisher Scientific	Cat#10407773 CAS: 25322-68-3
ProLong™ Gold Antifade Mountant	Thermo Fisher	Cat# P36934
cOmplete mini EDTA-free protease inhibitor cocktail tablets	Roche	Cat# 11836170001
Pierce™ Protease Inhibitor Mini Tablets, EDTA-free	Thermo Fisher	Cat#A32955
Restore™ Western Blot Stripping Buffer	Thermo Fisher	Cat# 21059
Sephadex G-25 resin	Merck	Cat# G2580 CAS: 9041-35-4

(*Continued on next page*)

Continued

REAGENT or RESOURCE	SOURCE	IDENTIFIER
Streptavidin-horseradish peroxidase	Thermo Fisher	Cat#43-4323
Tris(2-carboxyethyl)phosphine hydrochloride (TCEP HCl)	Fluorochem	Cat# M02624-10G<br>CAS: 51805-45-9
Triton™ X-100	Merck	Cat#T8787 CAS:9002-93-1
Tween-20	Merck	Cat#7949 CAS:9005-64-5
jetPRIME® DNA and siRNA transfection reagent	VWR	Cat#114-07
**Critical commercial assays**		
GeneMorph II Random Mutagenesis kit	Agilent	Cat# 200550
Q5® Site-Directed Mutagenesis Kit	New England Biolabs	E0554S
Pierce™ BCA Protein Assay Kit	Thermo Fisher	Cat#23227
**Deposited data**		
pDEST14-SpyCatcher	Zakeri et al., 2012	GenBank JQ478411
pDEST14-DogCatcher	This paper	GenBank MZ365292
pDEST14-SpyCatcher003	Keeble et al., 2019	GenBank MN433887
pET28a-SpyTag003-MBP	Keeble et al., 2019	GenBank MN433888
pET28-AviTag-DogTag-MBP	This paper	GenBank MZ365293
pET28a-HaloTag7SS-DogTag	Buldun et al., 2018	GenBank MZ365294
pET28-Gre2p-SpyTag003 Loop A	This paper	GenBank MZ365295
pET28-Gre2p-SpyTag003 Loop B	This paper	GenBank MZ365296
pET28-Gre2p-SpyTag003 Loop C	This paper	GenBank MZ365297
pET28-Gre2p-DogTag Loop A	This paper	GenBank MZ365298
pET28-Gre2p-DogTag Loop B	This paper	GenBank MZ365299
pET28-Gre2p-DogTag Loop C	This paper	GenBank MZ365300
pET28-sfGFP-SpyTag003 Loop A	This paper	GenBank MZ365301
pET28- sfGFP-SpyTag003 Loop B	This paper	GenBank MZ365302
pET28-sfGFP-SpyTag003 Loop C	This paper	GenBank MZ365303
pET28-sfGFP-DogTag Loop A	This paper	GenBank MZ365304
pET28- sfGFP-DogTag Loop B	This paper	GenBank MZ365305
pET28-sfGFP-DogTag Loop C	This paper	GenBank MZ365306
pET28-MBP-sTEV	This paper	GenBank MZ365307
pET28-SnoopCatcher	Veggiani et al., 2016	GenBank KU500646
pET28-AviTag-DogCatcher-MBP	This paper	GenBank MZ365308
pDEST14-Cys-DogCatcher	This paper	GenBank MZ365309
pJ404-SpyCatcher003-sfGFP	Keeble et al., 2019	GenBank MN433889
pJ404-DogCatcher-sfGFP	This paper	GenBank MZ365300
pcDNA4-TRPC5-SYFP2	Minard et al., 2019 This paper	GenBank MZ223439
pcDNA4-TRPC5-DogTag-SYFP2	This paper	GenBank MZ223440
Crystal structure of the pilus adhesin RrgA	Izore et al., 2010	PDB 2WW8
Crystal structure of HaloTag	Kang et al., 2017	PDB 5Y2X
Crystal structure of a superfolder green fluorescent protein	Pedelacq et al., 2006	PDB 2B3P
Crystal structure of yeast isovaleraldehyde reductase Gre2 complexed with NADPH	Guo et al., 2014	PDB 4PVD
Crystal structure of the SpyTag/SpyCatcher complex	Li et al., 2014	PDB 4MLI
Crystal structure of the *Streptococcus pyogenes* fibronectin binding protein Fbab-B	Oke et al., 2010	PDB 2X5P
Crystal structure of human TRPC5	Wright et al., 2020	PDB 6YSN
**Experimental models: cell lines**		
COS-7	ATCC	Cat#CRL-1651
HEK 293	ATCC	Cat#CRL-1573

(*Continued on next page*)

Continued		
REAGENT or RESOURCE	SOURCE	IDENTIFIER
**Oligonucleotides**		
Primer: pFabVec-fwd: forward primer: 5'-GGATCCAGTGGTAGCGAAAACCTCTAC	This paper	N/A
Primer: pFabVec-rev: 5'-CATGGCGCCCTGATCTCGAGG	This paper	N/A
Primer R2CatIns-fwd: 5'-GACCTCGAGATCAGGGCGCCATG	This paper	N/A
Primer R2CatIns-rev: 5'-GAAGTAGAGGTTTTCGCTACCACTGGATC	This paper	N/A
**Recombinant DNA**		
pDEST14-R2Catcher	This paper	N/A
pDEST14-SpyCatcher	Zakeri et al., 2012	Addgene Cat#35044
pFab5cHis-R2CatcherB-gIII	This paper	N/A
pDEST14-R2CatcherB	This paper	N/A
pDEST14-DogCatcher	This paper	Addgene Cat#171772
pDEST14-SpyCatcher003	Keeble et al., 2019	Addgene Cat# 133447
pET28a-His$_6$-MBP	Keeble et., 2017	N/A
pET28-AviTag-R2Tag-MBP	This paper	N/A
pET28a-SpyTag003-MBP	Keeble et al., 2019	Addgene Cat#133450
pET28-AviTag-DogTag-MBP	This paper	Addgene Cat#171773
pET28-AviTag-DogTag NA-MBP	This paper	N/A
pET28a-HaloTag7SS-DogTag	Buldun et al., 2018	Addgene Cat#171775
pET28-SpyTag003-sfGFP	Keeble et al., 2019	Addgene Cat#133454
pET28-Gre2p	This paper	N/A
pET28-Gre2p-SpyTag003 Loop A	This paper	N/A
pET28-Gre2p-SpyTag003 Loop B	This paper	N/A
pET28-Gre2p-SpyTag003 Loop C	This paper	N/A
pET28-Gre2p-DogTag Loop A	This paper	N/A
pET28-Gre2p-DogTag Loop B	This paper	N/A
pET28-Gre2p-DogTag Loop C	This paper	N/A
pET28-sfGFP-SpyTag003 Loop A	This paper	Addgene Cat#171776
pET28- sfGFP-SpyTag003 Loop B	This paper	Addgene Cat# 171777
pET28-sfGFP-SpyTag003 Loop C	This paper	Addgene Cat# 171778
pET28-sfGFP-DogTag Loop A	This paper	Addgene Cat# 171779
pET28- sfGFP-DogTag Loop B	This paper	Addgene Cat# 171780
pET28-sfGFP-DogTag Loop C	This paper	Addgene Cat# 171781
pET28-MBP-sTEV	This paper	Addgene Cat# 171782
pET28-SnoopCatcher	Veggiani et al., 2016	Addgene Cat#72322
pET28-Affi-SnoopCatcher	This paper	N/A
pET28-SnoopTagJr-AffiHer2	Buldun et al., 2018	N/A
pET28-AviTag-DogCatcher-MBP	This paper	Addgene Cat# 171928
pDEST14-Cys-DogCatcher	This paper	Addgene Cat# 171929
pJ404-SpyCatcher003-sfGFP	Keeble et al., 2019	Addgene Cat# 133449

(*Continued on next page*)

Continued

REAGENT or RESOURCE	SOURCE	IDENTIFIER
pJ404-DogCatcher-sfGFP	This paper	Addgene Cat# 171930
pGEX-2T-GST-BirA	Fairhead and Howarth, 2015	N/A
pcDNA4-TRPC5-SYFP2	Minard et al., 2019 This paper	N/A
pcDNA4-TRPC5-DogTag-SYFP2	This paper	N/A
Software and algorithms		
SoftMax Pro 7 software (Version 7.0.3; for Flexstation 3)	Molecular Devices	https://www.moleculardevices.com/
GeneSys software (Version 1.7.2.0; for Syngene G:BOX imager)	Syngene	https://www.syngene.com/support/software-downloads/
Fiji (ImageJ Version 1.53c)	ImageJ	https://imagej.net/Fiji
Zen Black 2010	Carl Zeiss Ltd.	
CamSol	Sormanni et al., 2015	https://www-cohsoftware.ch.cam.ac.uk/index.php
FluorEssence V3.5	Horiba-Yvon	https://www.horiba.com
Image Gauge version v4.21	Fujifilm	https://www.fujifilm.com
Image Lab 5.2.1	Bio-Rad	https://www.bio-rad.com
MassHunter Quantitative Analysis software version 7.0	Agilent	https://www.agilent.com/en/product/software-informatics/mass-spectrometry-software
MOLPROBITY	Lovell et al., 2003	http://kinemage.biochem.duke.edu
ProtParam	Gasteiger et al., 2005	https://web.expasy.org/protparam/
PyMOL version 2.0.6	DeLano Scientific/Schrodinger	https://pymol.org/2/
Rosetta 3 suite	Rosetta Commons	https://www.rosettacommons.org/
V-550 Spectra Manager	Jasco	https://jascoinc.com/

RESOURCE AVAILABILITY

Lead contact

Further information and requests for resources and reagents should be directed to and will be fulfilled by the lead contact, Mark Howarth (mark.howarth@bioch.ox.ac.uk).

Material availability

Requests for plasmids generated in this study which are not deposited in Addgene (listed here in the key resources table) should be directed to the Lead Contact, Mark Howarth (mark.howarth@bioch.ox.ac.uk), except for enquiries for TRPC5 reagents, which should be directed to r.bon@leeds.ac.uk.

Data and code availability

The sequences of relevant constructs are available in GenBank as described in the Key Resources table. This paper does not report original code. Any additional information required to reanalyze the data reported in this paper is available from the lead contact upon request.

EXPERIMENTAL MODEL AND SUBJECT DETAILS

See Method Details below to find details on mammalian cell culture and bacterial culture conditions.

METHOD DETAILS

Bacterial strain

Plasmids used in the present study were amplified using either *E. coli* NEB Turbo cells or *E. coli* K12 ER2738 cells which were grown in LB medium at 37°C. Proteins were expressed in *E. coli* BL21 (DE3) RIPL or *E. coli* C41 (DE3) cells, which were grown in LB medium +0.8% (w/v) glucose. Phage production for phage display selections were carried out using *E. coli* K12 ER2738 cells grown in 2YT media.

Cell lines

HEK 293 and COS-7 cells (both from ATCC, Teddington, UK) were maintained in Dulbecco's Modified Eagle Medium (Thermo Fisher), supplemented with 10% fetal bovine serum (Merck) and 100 units/mL penicillin with 100 μg/mL streptomycin (Thermo Fisher), in a humidified incubator at 37°C in 95% (v/v) air and 5% (v/v) CO_2.

Cloning of constructs

PCR-based cloning and site-directed mutagenesis were carried out using Q5 High-Fidelity Polymerase (NEB) or KOD polymerase (EMD Millipore) and Gibson assembly. pDEST14-R2Catcher was derived by cloning residues 734–838 of the RrgA adhesin from *Streptococcus pneumoniae* TIGR4 (GenBank AAK74622), with numbering based on PDB ID 2WW8 (Izore et al., 2010) into the backbone from pDEST14-SpyCatcher (Zakeri et al., 2012). Mutations D737E, N744D, N746T, N780D, K792T, A808P and N825D were overlaid on to R2Catcher to form pDEST14-R2CatcherB by Gibson assembly. Phagemid vector pFab5cHis-R2CatcherB-gIII was derived from pFab5cHis-SpyCatcher-gIII (Keeble et al., 2017). pDEST14-DogCatcher (Figure S2A) was derived from pDEST14-R2CatcherB by inclusion of the F802I, A820S and Q822R mutations by Gibson assembly. pDEST14-SpyCatcher003 has been described (Keeble et al., 2019). pET28a-His_6-MBP, encoding a His_6-tag linked to *E. coli* maltose binding protein (MBP) was described previously (Keeble et al., 2017). pET28-AviTag-R2Tag-MBP (Figure S2B) was derived from pET28a-SpyTag003-MBP (Keeble et al., 2019). pET28-AviTag-DogTag-MBP (Figure S2B) was derived from pET28a-SpyTag003-MBP (Keeble et al., 2019). pET28-AviTag-DogTag NA-MBP (the non-reactive N854A mutant) was derived from pET28-AviTag-DogTag-MBP by Gibson assembly. pET28a-HaloTag7SS-DogTag, with DogTag inserted in HaloTag7 between residues D139 and E140 and C61S and C261S mutations in HaloTag7 to block disulfide bond formation was previously described (Buldun et al., 2018). pET28-Gre2p was derived from pET28-SpyTag003-sfGFP (Keeble et al., 2019) by inserting the Gre2p isovaleraldehyde reductase from *Saccharomyces cerevisiae* (as a synthetic gene block with codons optimized for expression in *E. coli* B strains) in place of sfGFP by Gibson assembly. pET28-Gre2p-SpyTag003 loop insertions were derived from pET28-Gre2p by insertion of spacer-SpyTag003-spacer (sequence GGGGSRGVPHIVMVDAYKRYKGGGGS) between residues Lys140 and Ser141 (pET28-Gre2p-SpyTag003 Loop A), Glu229 and Asp230 (pET28-Gre2p-SpyTag003 Loop B), or Ser297 and Thr303 (pET28-Gre2p-SpyTag003 Loop C) by Gibson assembly. pET28-Gre2p-DogTag loop insertions were derived from pET28-Gre2p by insertion of spacer-DogTag-spacer (sequence GGGGSDIPATYEFTDGKHYITNEPIPPKGGGGS) between residues Lys140 and Ser141 (pET28-Gre2p-DogTag Loop A), Glu229 and Asp230 (pET28-Gre2p-DogTag Loop B), or Ser297 and Thr303 (pET28-Gre2p-DogTag Loop C) by Gibson assembly. pET28-sfGFP was derived from pET28-SpyTag003-sfGFP (Addgene plasmid ID 133454) (Keeble et al., 2019) by deletion of the N-terminal SpyTag003 by Gibson assembly. pET28-sfGFP-SpyTag003 loop insertions were derived from pET28-sfGFP by insertion of spacer-SpyTag003-spacer (sequence GGGGSRGVPHIVMVDAYKRYKGGGGS) between residues Val22 and Asn23 (pET28-sfGFP-SpyTag003 Loop A), Asp102 and Asp103 (pET28-sfGFP-SpyTag003 Loop B), or Asp173 and Gly174 (pET28-sfGFP-SpyTag003 Loop C) by Gibson assembly. pET28-sfGFP-DogTag loop insertions were derived from pET28-sfGFP by insertion of spacer-DogTag-spacer (sequence GGGGSDIPATYEFTDGKHYITNEPIPPKGGGGS) between Val22 and Asn23 (pET28-sfGFP-DogTag Loop A), Asp102 and Asp103 (pET28-sfGFP-DogTag Loop B), or Asp173 and Gly174 (pET28-sfGFP-DogTag Loop C) by Gibson assembly. pGEX-2T-GST-BirA was a gift from Chris O'Callaghan, University of Oxford. pET28-MBP-sTEV is a modified TEV protease construct with the domain arrangement MBP-His_6-TEV protease-Arg_6, modified from a kind gift of Stephen Bottomley, Monash University, but with no internal TEV cleavage site between the MBP and TEV protease. The TEV protease domain contains the following solubility/stability mutations (numbers refer to the standard TEV protease numbering scheme): C19V L56V C110V C130S S135G and S219D (Cabrita et al., 2007; Correnti et al., 2018; Kapust et al., 2001). pET28 Affi-SnoopCatcher was created by cloning an anti-HER2 affibody on to the N-terminus of pET28 SnoopCatcher (Veggiani et al., 2016). pET28-SnoopTagJr-AffiHer2 was previously described (Buldun et al., 2018). pET28-AviTag-DogCatcher-MBP was derived from pET28-AviTag-DogTag-MBP and pDEST14-DogCatcher by Gibson assembly. pDEST14-Cys-DogCatcher was derived by Gibson assembly from pDEST14-DogCatcher by insertion of a cysteine between the TEV cleavage site and the DogCatcher portion. pJ404-DogCatcher-sfGFP was derived by incorporating DogCatcher in place of SpyCatcher003 in pJ404-SpyCatcher003-sfGFP (Keeble et al., 2019).

pcDNA4-TRPC5-SYFP2 with human TRPC5 fused to SYFP2 has been described (Minard et al., 2019). For pcDNA4-TRPC5-DogTag-SYFP2, DogTag (underlined), flanked by a glycine-serine linkers (GGGGSDIPATYEFTDGKHYITNEPIPPKGGGGS) was introduced between Y460 and N461 of human TRPC5 by site-directed mutagenesis with Q5 High-Fidelity DNA polymerase (NEB). All constructs were verified by sequencing.

Protein expression and purification

R2Catcher, DogCatcher variants, AviTag-R2Tag-MBP, DogTag-MBP fusions, SpyTag003-MBP, SpyCatcher003-sfGFP and His_6-MBP were expressed in *E. coli* BL21 DE3 RIPL (Agilent). SpyCatcher003 was expressed in *E. coli* C41 DE3 (a gift from Anthony Watts, University of Oxford). Single colonies were inoculated into 10 mL LB containing either 100 μg/mL ampicillin (SpyCatcher003, SpyCatcher003-sfGFP, R2Catcher or DogCatcher variants) or 50 μg/mL kanamycin (His_6-MBP, SpyTag003-MBP, AviTag-R2Tag-MBP and DogTag fusions) and grown for 16 hr at 37°C with shaking at 200 rpm. For secondary culture, 1/100 dilution of the saturated overnight culture was inoculated in 1 L LB + 0.8% (w/v) glucose with appropriate antibiotic and grown at 37°C with shaking at 200 rpm in ultra-yield baffled flasks (Thomson Instrument Company) until an OD_{600} of 0.5, followed by induction with 0.42 mM IPTG at 30°C with shaking at 200 rpm for 4 hr. Cells were harvested and then lysed by sonication on ice in Ni-NTA buffer (50 mM Tris-HCl pH 8.0 containing 300 mM NaCl) and 10 mM imidazole with mixed protease inhibitors (cOmplete mini EDTA-free protease inhibitor cocktail,

Roche) and 1 mM phenylmethylsulfonyl fluoride (PMSF), followed by clarification by centrifugation in an JA25−50 rotor (Beckman) at 30,000–35,000 g for 30–40 min at 4°C. The clarified lysate was incubated with Ni-NTA resin (Qiagen). After addition of the resin/lysate slurry to a Poly-Prep gravity column, the resin was washed with 30 column volumes of Ni-NTA buffer containing 10 mM imidazole, followed by elution using N-NTA buffer containing 200 mM imidazole (Fairhead and Howarth, 2015). Proteins were dialyzed into PBS (137 mM NaCl, 2.7 mM KCl, 10 mM Na_2HPO_4, 1.8 mM KH_2PO_4) pH 7.5 using 3.5 kDa molecular weight cut-off dialysis tubing (Spectrum Labs). MBP-sTEV was expressed and purified as described above except without protease inhibitor cocktail tablets. Protein concentrations were determined from OD_{280} using the extinction coefficient from ExPASy ProtParam.

GST-BirA was expressed in *E. coli* BL21 DE3 RIPL as above and purified using glutathione Sepharose (Fairhead and Howarth, 2015). Variants of sfGFP were expressed in *E. coli* BL21 DE3 RIPL and purified as above, except after induction the culture was grown at 22°C for 18 hr. Variants of Gre2p were expressed in *E. coli* BL21 DE3 RIPL and purified as above, except after induction the culture was grown at 25°C for 18 hr and proteins were dialyzed into 100 mM potassium phosphate pH 7.4 [formed by mixing 100 mM solutions of monobasic (KH_2PO_4) and dibasic (K_2HPO_4) potassium phosphate solutions]. Proteins were quantified using the Pierce bicinchoninic acid (BCA) Protein assay kit (Thermo Fisher) according to the manufacturer's instructions, with the modification that sfGFP variants were incubated for 1 hr at 60°C in the assay solution before reading the absorbance, to ensure complete denaturation.

Typical protein yields per L of culture were: R2Catcher 4 mg, DogCatcher variants 6–8 mg, Tag-MBP fusions 20–25 mg, sfGFP fusions 15–35 mg, Gre2p fusions 12–24 mg.

AviTag biotinylation with GST-BirA was performed as described (Fairhead and Howarth, 2015): a master mix was made of 100 μM target protein in 952 μL PBS, 5 μL 1 M $MgCl_2$, 20 μL 100 mM ATP, 20 μL 50 μM GST-BirA and a final concentration of 1.5 mM biotin. The reaction was incubated for 1 hr at 30°C with shaking at 800 rpm. An additional 20 μL 50 μM GST-BirA was added, followed by a further 1 hr incubation. Finally, the sample was dialyzed in PBS pH 7.5 at 4°C. We established complete biotinylation by a streptavidin gel shift assay (Fairhead and Howarth, 2015).

R2CatcherB WT phage production

We chose two different cell-lines to identify better conditions for R2CatcherB phage production, since R2CatcherB initially displayed poorly on the phage surface. R2CatcherB phagemid was transformed into *E. coli* XL1-Blue (Agilent) or *E. coli* K12 ER2738 (Lucigen) and grown at 18, 25 or 30°C for 16 hr for phage production. The ER2738 strain was preferred, giving increased functionality of phage for the selection. Transformed cells were grown in 50 mL 2YT with 100 μg/mL ampicillin, 10 μg/mL tetracycline and 0.2% (v/v) glycerol at 37°C, 200 rpm until OD_{600} = 0.5 (~2–3 hr). Cells were infected in log phase with 10^{12} R408 helper phage (Agilent) and incubated at 80 rpm at 37°C for 30 min. Expression of R2CatcherB-pIII was induced with 0.1 mM IPTG and cells were incubated for 18–20 hr at 200 rpm at 18, 25 or 30°C. Phage were harvested using one volume of precipitation buffer [sterile 20% (w/v) PEG8000, 2.5 M NaCl] per 4 volumes of supernatant (Keeble et al., 2017). Briefly, the supernatants were mixed with the precipitation buffer and incubated at 4°C for 3–4 hr. Phage were pelleted by centrifugation at 15,000 g for 30 min at 4°C and the supernatant was removed. Phage pellets were resuspended in PBS (2 mL per 100 mL culture) and centrifuged at 15,000 g for 10 min at 4°C to clear any residual cells, before the supernatant was transferred to a new tube. The mixture was precipitated again as previously, but this time resuspended in 0.25 mL PBS per 100 mL culture. Samples were centrifuged at 15,000 g for 10 min at 4°C and phage were precipitated a third time and resuspended in a final volume of 0.25 mL PBS per 100 mL culture. Samples were stored short-term (1–2 weeks) at 4°C, or long-term at −80°C with 20% (v/v) glycerol as cryoprotectant. Phage were quantified by plating serial dilutions after re-infection.

Phage library generation

To create the randomized mutagenesis library, pFab5cHis-R2CatcherB-gIII phagemid was used as a template in PCR reactions. The vector was amplified using KOD polymerase (EMD Millipore) with oligonucleotide primers (forward primer: 5′-GGATCCAGTGGTA GCGAAAACCTCTAC; reverse primer: 5′-CATGGCGCCCTGATCTCGAGG). The insert was amplified with forward primer 5′- GACC TCGAGATCAGGGCGCCATG and reverse primer 5′- GAAGTAGAGGTTTTCGCTACCACTGGATC using GeneMorph II Random Mutagenesis kit (Agilent) according to the manufacturer's protocol. DpnI was added following thermal cycling, incubated at 37°C for 1 hr, and heat-inactivated at 80°C for 20 min. The amplified fragments were separated by agarose gel electrophoresis and DNA bands for the vector and insert were purified by gel extraction (Thermo Scientific). Ligation was performed at the optimized vector:insert molar ratio of 1:3 with ~500 ng of DNA in a total volume of 20 μL. Equal volume of 2× master mix Gibson (NEB) was added to the insert-vector mixture and incubated at 50°C for 16 hr. DNA was concentrated on a spin-filter (Wizard PCR clean up kit; Promega) and 3 μL (~700 ng) of DNA was transformed into 50 μL electrocompetent ER2738 amber stop codon suppressor cells (Lucigen) by electroporation in Bio-Rad 2 mm electroporation cuvettes in a Gene Pulser Xcell (Bio-Rad) with a 2.5 kV voltage setting. Transformants were recovered by addition of 950 μL SOC medium at 37°C for 1 hr and then further grown in 50 mL 2YT media, containing 100 μg/mL ampicillin and 10 μg/mL tetracycline for 16 hr at 37°C. Transformation efficiency was determined by plating serial dilutions of 1 mL rescue culture on an agar plate with 100 μg/mL ampicillin and 10 μg/mL tetracycline. Aliquots were flash-frozen and stored at −80°C. To harvest the library, 1 mL of overnight culture was added to 250 mL 2YT media with 100 μg/mL ampicillin and 10 μg/mL tetracycline and 0.2% (v/v) glycerol and grown at 37°C at 200 rpm until OD_{600} 0.5 (~2–3 hr). Cells were infected with 10^{12} R408 helper phage (Agilent) and incubated at 80 rpm at 37°C for 30 min. Expression of R2CatcherB-pIII library was induced with 0.1 mM IPTG and incubated for 18–20 hr at 200 rpm at 18°C. Cells were removed by centrifugation at 15,000 g for 10 min at 4°C and phage were purified as described above.

Phage selections

Biotinylated AviTag-DogTag-MBP was used as bait to react with the R2CatcherB phage library. The non-reactive bait variant (biotinylated AviTag-DogTag NA-MBP) was included in parallel selections to assess the efficiency of the panning. Reactions were carried out in PBS pH 7.5 at 25°C with 3% (w/v) BSA (BSA, Merck A9418) and supplemented with 25 μM His_6-MBP (to counter-select for any DogCatcher variants that bind to MBP). In the first round of selection, 10^{12} phage were mixed with 0.5 μM bait and reacted for 18 hr. Three subsequent selection rounds were carried out with increasing stringency (0.2 μM bait and 60 min reaction in round 2; 0.1 μM bait and 15 min reaction in round 3; 0.05 μM bait and 10 min reaction in round 4). Reaction was stopped by adding 100-fold excess bait without an AviTag (DogTag-MBP).

Phage were purified from unreacted biotinylated bait by PEG-NaCl precipitation. The pellet containing the phage-biotinylated bait adduct was resuspended in PBS pH 7.5 with 0.1% (v/v) Tween 20. 200 μL phage were mixed with 20 μL Biotin-Binder Dynabeads (Thermo Fisher) in a 96-well low bind Nunc plate that had been pre-blocked for 2 hr at 25°C with 3% (w/v) BSA in PBS pH 7.5 + 0.1% (v/v) Tween 20. The beads were pre-washed four times with 200 μL/well of PBS pH 7.5 + 0.1% (v/v) Tween 20. Phage-biotinylated bait adduct was incubated with beads in the microtiter plate for 1 hr at 25°C with shaking at 800 rpm in an Eppendorf Thermomixer. To remove weakly bound phage, beads were washed once with 150 μL glycine-HCl pH 2.2 at 25°C, then four times with 150 μL TBS (50 mM Tris-HCl + 150 mM NaCl, pH 7.5) with 0.5% (v/v) Tween 20 at 25°C. Phage were eluted from beads with 100 μL 0.72 mg/mL MBP-sTEV at 34°C for 2 hr in 50 mM Tris-HCl pH 8.0 with 0.5 mM ethylenediamine tetraacetic acid (EDTA). Eluted phage were rescued by infection of 10 mL mid-log phase (OD_{600} = 0.5) cultures of ER2738 cells. Cells were grown at 37°C at 80 rpm for 30 min and then transferred into 200 mL 2YT supplemented with ampicillin (100 μg/mL), tetracycline (10 μg/mL), 0.2% (v/v) glycerol and grown at 37°C at 200 rpm for ~2 hr until OD_{600} = 0.5. Cultures were infected with 10^{12} R408 helper phage and incubated at 80 rpm at 37°C for 30 min. Expression of R2CatcherB-pIII was induced with 0.1 mM IPTG and cells were incubated for 18–20 hr at 200 rpm at 18°C. The number of phage eluted was quantified by plating serial dilutions from 10 mL rescue culture.

Isopeptide bond formation assays

Reactions were generally carried out at 25°C in PBS pH 7.5 (Keeble et al., 2017). Reactions were analyzed by SDS-PAGE on 16% (w/v) polyacrylamide gels using the XCell SureLock system (Thermo Fisher) at 180 V. The reaction was quenched by addition of 6× SDS-loading buffer [0.23 M Tris-HCl, pH 6.8, 24% (v/v) glycerol, 120 μM bromophenol blue, 0.23 M SDS] and heating at 95°C for 5 min in a Bio-Rad C1000 thermal cycler. Proteins were stained using InstantBlue (Expedeon) Coomassie. Band intensities were quantified using a Gel Doc XR imager and Image Lab 5.0 software (Bio-Rad). Percentage isopeptide bond formation was calculated by dividing the intensity of the band for the covalent complex by the intensity of all the bands in the lane and multiplying by 100.

The second-order rate constant for covalent complex formation when reacting 5 μM AviTag-DogTag-MBP and 5 μM Catcher protein was determined by monitoring the reduction in the relative intensity of the band for R2Catcher or DogCatcher, to give the change in the concentration of the unreacted Catcher variant. Time-points were analyzed during the linear portion of the reaction curve. 1/[Catcher variant] was plotted against time and analyzed by linear regression using Excel (Microsoft) and Origin 2015 (OriginLab Corporation), including calculation of the s.d. for the best fit. The data represent the mean ±1 s.d. from triplicate measurement.

Temperature-dependence of DogTag:DogCatcher isopeptide bond formation was carried out in succinate–phosphate–glycine (SPG) buffer (12.5 mM succinic acid, 43.75 mM NaH_2PO_4, 43.75 mM glycine; pH adjusted to 7.0 using NaOH) with 2 μM of AviTag-DogTag-MBP and DogCatcher with the 15 min time point assessed at 4, 25 or 37°C in triplicate.

The pH-dependence of DogTag:DogCatcher isopeptide bond formation was carried out in SPG buffer with 2 μM each for AviTag-DogTag-MBP and DogCatcher with the 30 min time point assessed at pH 4, 5, 6, 7, 8, or 9 in triplicate.

The buffer-dependence of DogTag:DogCatcher isopeptide bond formation was carried out in a range of buffers all at pH 7.5 with 5 μM AviTag-DogTag-MBP and 5 μM DogCatcher with the 5 min time point assessed. Buffers used were PBS, PBS +1 mM dithiothreitol (DTT), PBS +1 mM EDTA, PBS +1% (v/v) Triton X-100, PBS +1% (v/v) Tween 20, HBS (50 mM HEPES +150 mM NaCl), TBS (50 mM Tris-HCl + 150 mM NaCl), or Tris (50 mM Tris-HCl).

Condition-dependence of SpyTag003/SpyCatcher003 was determined as follows. For the temperature-dependence assay, 100 nM SpyCatcher003-sfGFP and SpyTag003-MBP were reacted for 2 min in PBS pH 7.4 supplemented with 0.2% (w/v) BSA at 4, 25, 30 or 37°C. For the buffer-dependence assay, 100 nM SpyCatcher003-sfGFP and SpyTag003-MBP were reacted for 2 min at 25°C in PBS pH 7.4, PBS pH 7.4 + 1 mM EDTA, PBS pH 7.4 + 1% (v/v) Triton X-100, PBS pH 7.4 + 1% (v/v) Tween 20, HBS (20 mM HEPES pH 7.4 + 150 mM NaCl), or TBS (20 mM Tris-HCl pH 7.4 + 150 mM NaCl). Each buffer was supplemented with 0.2% (w/v) BSA. For the pH-dependence assay, 1 μM SpyCatcher003 and 1 μM SpyTag003-MBP were reacted in SPG buffer at 25°C.

DogCatcher and DogTag reaction toward completion was tested with 10 or 20 μM DogCatcher reacting with 10 or 20 μM HaloTag7SS-DogTag in PBS pH 7.5 at 25°C for 200 min. 5 μM DogCatcher was reacted with either 5 μM HaloTag7SS-DogTag or AviTag-DogTag-MBP in PBS pH 7.5 at 25°C, to compare the reaction of DogTag constrained in a loop (HaloTag7SS-DogTag) or free from this constraint (AviTag-DogTag-MBP).

Reaction of loop variants for sfGFP or Gre2p was carried out in PBS pH 7.5 at 25°C with 5 μM loop variant reacted with 5 μM DogCatcher or SpyCatcher003.

Reaction at low concentration was carried out with 100 nM DogCatcher-sfGFP and 100 nM HaloTag7SS-DogTag in PBS pH 7.5 + 0.2% (w/v) BSA (Merck) at 25°C. Reactions were analyzed by SDS/PAGE on 16% polyacrylamide gels using the XCell

SureLock system (Thermo Fisher) at 180 V. The reaction was quenched at 50°C for 5 min after addition of one-sixth the volume of 6× SDS-loading buffer in a Bio-Rad C1000 thermal cycler to retain the fluorescence of sfGFP. sfGFP fluorescence in gels was quantified using a Fluorescent Image Analyzer FLA-3000 (FujiFilm) and ImageGauge version 5.21 software. % Product was calculated by dividing the intensity of the band for the covalent complex by the intensity of all of the bands in the lane and multiplying by 100.

Cross-reactivity of DogCatcher (15 μM) and HaloTag7SS-DogTag (10 μM) was tested with Affi-SnoopCatcher, SnoopTagJr-AffiHer2, SpyCatcher003, SpyTag003-MBP (all at 10 μM for testing DogCatcher reactivity; with Affi-SnoopCatcher and SpyCatcher003 at 15 μM for reaction with HaloTag7SS-DogTag) in PBS pH 7.5 at 25°C for 24 hr.

Protein yield and solubility determination

During purification from *E. coli* (carried out in duplicate), R2Catcher, R2CatcherB or DogCatcher was eluted in Ni-NTA buffer containing 200 mM imidazole and centrifuged for 30 min at 17,000 g at 4°C. The concentration of the supernatant was measured by A_{280}. The proteins were dialyzed three times into PBS pH 7.5 at 4°C using 3.5 kDa molecular weight cut-off dialysis tubing (Spectrum Labs). A further 30 min centrifugation at 17,000 g at 4°C was carried out and the concentration of the supernatant was determined by A_{280}.

Proteins in PBS pH 7.5 were concentrated in a Vivaspin 6 (5,000 Da molecular weight cut-off, Cytiva) at 25°C at 4,000 g in a bench-top centrifuge with a swing-out rotor. The protein concentration was monitored periodically by A_{280}, with the onset of aggregation determined by the solution becoming visibly cloudy.

DogCatcher dye labeling

Dye labeling took place with tubes wrapped in foil, to minimize light exposure. Alexa Fluor 647-maleimide (Thermo Fisher) was dissolved in DMSO to 10 mg/mL. Cys-DogCatcher was dialyzed into TBS pH 7.4 and reduced for 30 min at 25°C with 1 mM TCEP [tris(2-carboxyethyl)phosphine)]. 100 μM Cys-DogCatcher was incubated with a 3-fold molar excess of dye:protein and reacted with end-over-end rotation at 25°C for 4 hr. After quenching the unreacted maleimide with 1 mM DTT for 30 min at 25°C, samples were centrifuged at 16,000 g for 5 min at 4°C to remove any aggregates. Free dye was removed using Sephadex G-25 resin (Merck) and dialyzing thrice each time for at least 3 hr in PBS pH 7.4 at 4°C.

Spectroscopic measurements of sfGFP

Emission spectra of 0.5 μM sfGFP variants were collected at 25°C in PBS pH 7.5, using a Horiba-Yvon Fluoromax 4 with an excitation wavelength of 488 nm. Fluorescence emission was collected between 500 and 660 nm using a monochromator, with data collected with polarizers set to the magic angle (54.7°). Absorbance spectra of 10 μM sfGFP variants were collected at 25°C in PBS pH 7.5 using a Jasco V-550 UV/VIS Spectrophotometer. Data were collected every nm from 250 nm to 600 nm with a scanning speed of 200 nm/min, a fast response, and a bandwidth of 2.0 nm. The data represent the mean of biological triplicates.

Mass spectrometry

30 μM DogCatcher was reacted with 15 μM DogTag peptide (GDIPATYEFTDGKHYITNEPIPPK; solid-phase synthesized by Activotec at >95% purity) for 2 hr in PBS pH 7.5 at 25°C, to enable pre- and post-reacted DogCatcher to be compared in a single experiment. Reaction was analyzed by SDS-PAGE with Coomassie staining. Mass spectrometry was performed using an Agilent RapidFire (RF365) fitted with high-throughput sampling robotic platform coupled to an Agilent 6550 Accurate-Mass Quadrupole Time-of-Flight (Q-TOF) mass spectrometer in positive ion mode, utilizing a jet-stream electrospray ion source (Agilent). 10 μM protein in 50 μL was prepared in a 384-well polypropylene plate (Greiner Bio-One) and then acidified by addition of 5 μL 10% (v/v) formic acid. Samples were aspirated from the plate under vacuum for 400 ms and loaded onto a C4 solid-phase extraction cartridge. The cartridge was washed with 0.1% (v/v) formic acid at 1.5 mL/min for 5.5 s. Proteins were eluted into the mass spectrometer using 85% (v/v) acetonitrile, 15% (v/v) deionized water containing 0.1% (v/v) formic acid at 1.25 mL/min for 5.5 s, with water used to re-equilibrate the cartridge for 500 ms. Nitrogen drying gas was operated at 13 L/min and 225°C. The jet stream sheath gas was 350°C and 12 L/min. The nozzle voltage was 1,500 V. MassHunter Quantitative Analysis software version 7.0 (Agilent) was used with the maximum entropy algorithm. Predicted mass was based on ExPASy ProtParam with removal of the initiating formyl methionine and loss of 17 Da upon formation of an isopeptide bond.

Gre2p activity assay

50 nM Gre2p variant was incubated with 1.5 mM isovaleraldehyde (Merck) and 0.25 mM reduced nicotinamide adenine dinucleotide phosphate (NADPH) (ChemCruz) in 100 mM potassium phosphate pH 7.4 [formed by mixing 100 mM solutions of monobasic (KH_2PO_4) and dibasic (K_2HPO_4) potassium phosphate solutions] + 0.1% (w/v) BSA +1 mM DTT at 25°C. Reaction was initiated by pipetting 100 μL 15 mM isovaleraldehyde in 100 mM potassium phosphate pH 7.4 into the reaction mixture and the progress was measured at 25°C by the decrease in A_{340}, measured using a Jasco V-550 UV/VIS Spectrophotometer with a medium response and 5.0 nm bandwidth. Data were collected every second for 200 s and represent the mean of 3 biological replicates. Specific activity was calculated by converting the change in absorbance with time to change in moles of NADPH with time, using an extinction coefficient for NADPH of 6,220 $M^{-1}cm^{-1}$. Values shown are the mean ±1 s.d. from three biological replicates.

Intracellular calcium measurement

HEK 293 cells were plated onto a 6-well plate at 0.8 × 10^6 cells/well for 24 hr prior to transfection. Cells were transfected with 2 μg DNA for either pcDNA4/TO (empty vector), TRPC5-SYFP2, or TRPC5-DogTag-SYFP2 using jetPRIME transfection reagent (VWR). 24 h after transfection, cells were plated onto black, clear-bottomed 96 well plates (Greiner) at 60,000 cells per well and left to adhere for 16–18 hr. For intracellular calcium recordings, media was removed and replaced with SBS containing 2 μM Fura-2 AM (Thermo Fisher) and 0.01% (v/v) pluronic acid. SBS contained (in mM): NaCl 130, KCl 5, glucose 8, HEPES 10, $MgCl_2$ 1.2, $CaCl_2$ 1.5, titrated to pH 7.4 with NaOH. Cells were then incubated for 1 hr at 37°C. After incubation, Fura-2 AM was removed and replaced with fresh SBS. Cells were incubated at 25°C for 30 min. SBS was then replaced with recording buffer [SBS with 0.01% (v/v) pluronic acid and 0.1% (v/v) DMSO, to match compound buffer]. For experiments to determine the effect of DogCatcher labeling on TRPC5 function, cells were washed twice with SBS after Fura-2 AM incubation. SBS with or without 5 μM biotin-DogCatcher-MBP was added and cells were incubated at 25°C for 30 min. The buffer was then replaced by recording buffer. Intracellular calcium was measured by use of a FlexStation3 (Molecular Devices), using excitation of 340 nm and 380 nm, with emission of 510 nm. Recordings were taken for 5 min at 5 s intervals. At 60 s, the agonist (−)-englerin A (PhytoLab) was added from a compound plate containing compound buffer [SBS with 0.01% (v/v) pluronic acid and (−)-englerin A] to a final concentration of 30 nM (Figure 7B) or 10 nM (Figure 7D).

GFP-trap and Western blot

The protocol is an adaptation of a previous photoaffinity labeling/GFP-Trap/Western blot workflow (Bauer et al., 2020). COS-7 cells were plated onto 6-well plates at 0.15 × 10^6 cells per well for 24 hr prior to transfection. Cells were transfected with 2 μg pcDNA4/TO (empty vector), pcDNA4-TRPC5-SYFP2, or pcDNA4-TRPC5-DogTag-SYFP2 using jetPRIME transfection reagent (VWR). After 4 hr, transfection media was replaced with fresh media. Experiments were carried out 24–48 hr after transfection. Media was removed and cells were washed once with PBS containing 1 mM $CaCl_2$. For the time course, biotin-DogCatcher-MBP was diluted to 5 μM using PBS containing 1 mM $CaCl_2$ and cells were incubated with biotin-DogCatcher-MBP for 1, 3, 10 or 30 min at 25°C on a rocker. Cells were washed three times with PBS containing 1 mM $CaCl_2$ and then lysed in lysis buffer [10 mM Tris-HCl pH 7.5, 150 mM NaCl, 0.5 mM EDTA, 0.5% (v/v) NP-40, supplemented with Pierce Protease Inhibitor Mini Tablets (Thermo Fisher)], for 30 min at 4°C. Lysates were centrifuged (12,000 g, 4°C, 10 min), and protein in supernatants was quantified by BCA assay (Thermo Fisher). Equal amounts of protein and lysis buffer were diluted in Dilution buffer (10 mM Tris-HCl pH 7.5, 150 mM NaCl, 0.5 mM EDTA) to 400 μL, and 30 μL was then removed for input blots. Washed GFP-Trap agarose (20 μL per reaction; Chromotek) was added to diluted lysates and incubated for 1 hr at 4°C on a rotator. GFP-Trap agarose was then washed three times with Dilution buffer and proteins were eluted with Novex Tris-Glycine loading buffer (2×; Thermo Fisher) supplemented with 10% (v/v) β-mercaptoethanol (Merck) at 95°C for 10 min. Prior to SDS-PAGE, GFP-Trap samples were centrifuged briefly to pellet the beads. GFP-Trap samples were separated on 7.5% pre-cast gels (Bio-Rad) and transferred to polyvinylidene difluoride (PVDF, Millipore). Membranes were blocked with 5% (w/v) milk in PBS supplemented with 0.1% (v/v) Tween 20 (PBS-T), before incubation with primary antibody (anti-GFP, Abcam ab1218; 1:5,000) overnight at 4°C. Following washing with PBS-T (6 × 5 min, 25°C) membranes were incubated with anti-mouse IgG-horseradish peroxidase (Thermo Fisher) for 1 hr at 25°C. Membranes were then washed with PBS-T (6 × 5 min, 25°C) and blots were imaged with Pierce ECL (Thermo Fisher) and a G:BOX imager with Syngene software. GFP-Trap membranes were then stripped with Restore Stripping Buffer (Thermo Fisher) and blocked in 5% (w/v) BSA in PBS-T, before incubation with streptavidin-horseradish peroxidase (1:5,000; Thermo Fisher) for 16–18 hr at 4°C. Blots were washed with PBS-T and imaged as above.

Fluorescence microscopy

COS-7 cells were plated onto sterile 13 mm glass coverslips at 40,000 cells per well in a 24 well plate. The next day, cells were transfected with 500 ng TRPC5-SYFP2 or TRPC5-DogTag-SYFP2 using jetPRIME transfection reagent (VWR). Transfection media was replaced with fresh media 4 hr after transfection. 24 h after transfection, cells were washed once with PBS containing 1% (w/v) BSA and incubated with 5 μM DogCatcher-647 in PBS for 1, 3, or 10 min at 25°C on a rocker in the dark. Cells were washed three times with PBS and fixed in 4% (w/v) paraformaldehyde (Alfa Aesar) in PBS for 10 min at 25°C, before being washed once in PBS +0.1 M glycine, pH 7.4. Cells were then incubated in fresh 0.1 M glycine in PBS at pH 7.4 for 10 min at 25°C. Cells were then washed three times with PBS and mounted with ProLong Gold (ThermoFisher Scientific). Imaging was carried out on a LSM710 confocal microscope (Zeiss) using a 63×/1.4 oil objective. SYFP2 was excited by a 514 nm Argon laser and Alexa Fluor 647 by a 633 nm HeNe laser. Fluorescence was detected using Zeiss photomultiplier tubes with wavelength ranges of 524–583 nm for SYFP2 and 660–718 nm for Alexa Fluor 647. Images were exported to Fiji (ImageJ) for final processing. The images represent confocal slices. All images were collected and analyzed using the same settings.

Structure visualization

Protein structures were rendered in PyMOL version 2.0.6 (DeLano Scientific), based on PDB 2WW8 (Izore et al., 2010), 5Y2X (Kang et al., 2017), 2B3P (Pedelacq et al., 2006), 4PVD (Guo et al., 2014), 4MLI (Li et al., 2014) or 6YSN (Wright et al., 2020). pI values for DogCatcher variants were predicted using ProtParam (Gasteiger et al., 2005).

QUANTIFICATION AND STATISTICAL ANALYSIS

Statistical analysis

Isopeptide bond formation assay measurements (rates and product yield at fixed time points) were means and standard deviations of triplicate experiments calculated using Excel (Microsoft) and Origin 2015 (OriginLab Corporation). Gre2p assay time courses and sfGFP spectra were means of triplicate biological replicates calculated using Excel (Microsoft). Gre2p assay rates were means and standard deviations of triplicate experiments calculated using Excel (Microsoft). The statistical details of experiments can be found in the Figure legends and STAR Methods.

Cell Chemical Biology, Volume *29*

Supplemental information

DogCatcher allows loop-friendly protein-protein ligation

Anthony H. Keeble, Vikash K. Yadav, Matteo P. Ferla, Claudia C. Bauer, Eulashini Chuntharpursat-Bon, Jin Huang, Robin S. Bon, and Mark Howarth

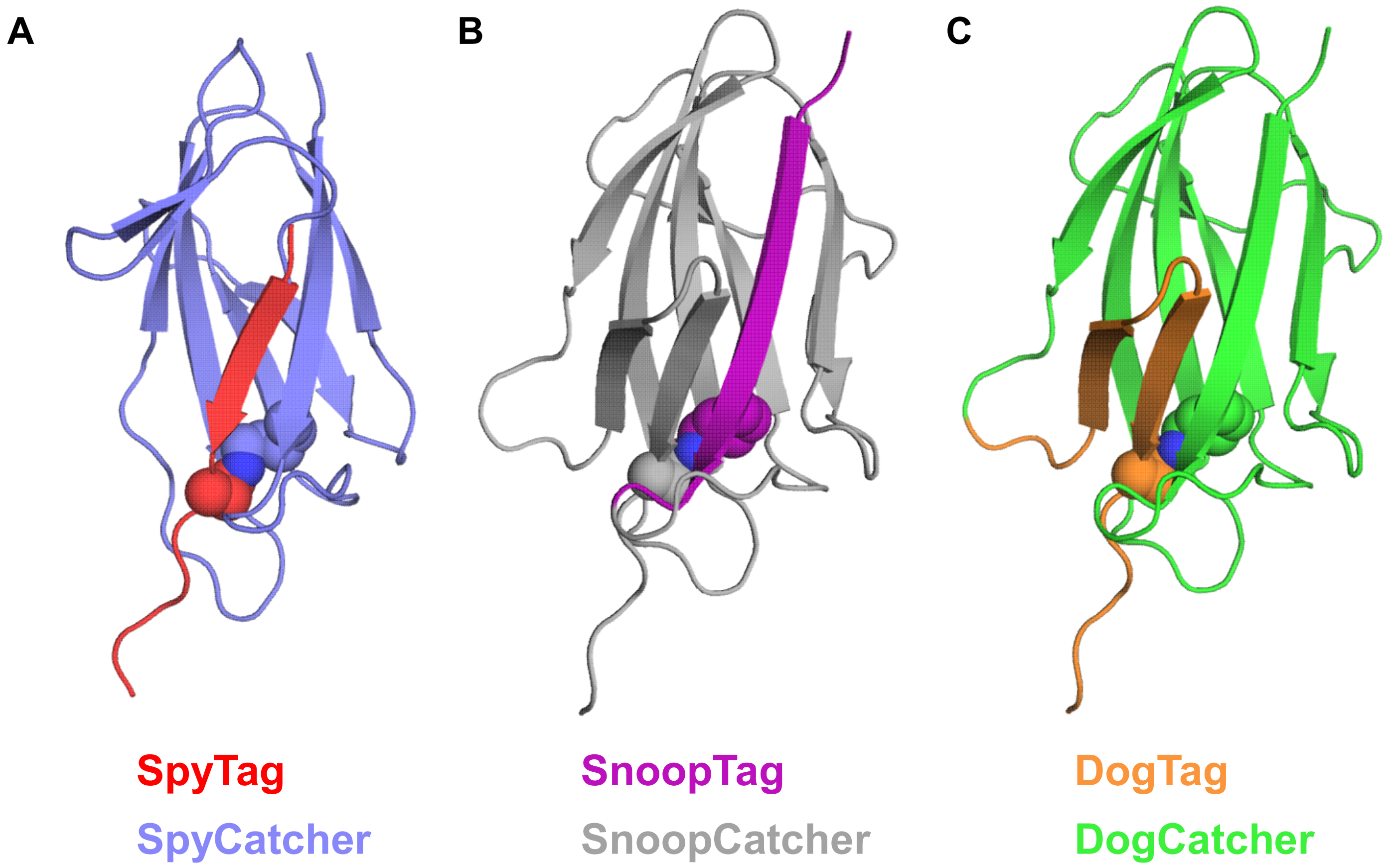

Figure S1: Structural comparison of the different Tag/Catcher pairs, related to Figure 1. **(A)** SpyTag/SpyCatcher has the Tag as a linear single β-strand (PDB: 2X5P and 4MLI). The isopeptide-forming residues of SpyTag (Asp117) and SpyCatcher (Lys31) are shown as spheres. **(B)** Model of SnoopTag/SnoopCatcher based on RrgA domain 4 (PDB: 2WW8). SnoopTag has the Tag derived from a linear single β-strand. The isopeptide-forming residues of SnoopTag (Lys742) and SnoopCatcher (Asn854) are shown as spheres. **(C)** Model of DogTag/DogCatcher based on RrgA domain 4 (PDB: 2WW8). DogTag/DogCatcher has the Tag derived from two β-strands arranged as a β-hairpin. The side-chains for the isopeptide-forming residues of the DogTag (Asn854) and DogCatcher (Lys742) are shown as spheres. The dark blue sphere indicates the nitrogen atom in the isopeptide bond.

A

```
                734  740        750        760        770        780
R2Catcher       KLGDIEFIKVNKNDKKPLRGAVFSLQKQHPDYPDIYGAIDQNGTYQNVRTGEDGK
DogCatcher      KLGEIEFIKVDKTDKKPLRGAVFSLQKQHPDYPDIYGAIDQNGTYQDVRTGEDGK

                 790        800        810        820        830
R2Catcher       LTFKNLSDGKYRLFENSEPAGYKPVQNKPIVAFQIVNGEVRDVTSIVPQ-
DogCatcher      LTFTNLSDGKYRLIENSEPPGYKPVQNKPIVSFRIVDGEVRDVTSIVPQ-
```

Reactive Lysine
Catalytic Glutamate
Mutations to create DogCatcher

B

```
                  840        850        860
R2Tag           DIPAGYEFTNDKHYITNEPIPPK
DogTag          DIPATYEFTDGKHYITNEPIPPK
```

Reactive Asparagine
Mutations to create DogTag

C

```
                734  740        750        760        770        780
Domain 4        KLGDIEFIKVNKNDKKPLRGAVFSLQKQHPDYPDIYGAIDQNGTYQNVRTGEDGK
SnoopCatcher                NDKKPLRGAVFSLQKQHPDYPDIYGAIDQNGTYQNVRTGEDGK
SnoopLigase              VNKNDKKPLRGAVFSLQKQHPDYPDIYGAIDQNGTYQNVRTGEDGK
DogCatcher      KLGEIEFIKVDKTDKKPLRGAVFSLQKQHPDYPDIYGAIDQNGTYQDVRTGEDGK

                 790        800        810        820        830        840
Domain 4        LTFKNLSDGKYRLFENSEPAGYKPVQNKPIVAFQIVNGEVRDVTSIVPQDIPAGY
SnoopCatcher    LTFKNLSDGKYRLFENSEPAGYKPVQNKPIVAFQIVNGEVRDVTSIVPQDIPATY
SnoopLigase     LTFKNLSDGKYRLFENSEPPGYKPVQNKPIVAFQIVNGEVRDVTSIVPPGVPATY
DogCatcher      LTFTNLSDGKYRLIENSEPPGYKPVQNKPIVSFRIVDGEVRDVTSIVPQ-

                      850        860
Domain4         EFTNDKHYITNEPIPPK-
SnoopCatcher    EFTNGKHYITNEPIPPK-
SnoopLigase     EFT-
DogCatcher
```

Reactive Lysine
Catalytic Glutamate
Reactive Asparagine

Figure S2: Amino acid sequences of the key protein variants, related to Figure 1. (A) Sequence alignment of R2Catcher with DogCatcher. **(B)** Sequence alignment of R2Tag with DogTag. **(C)** Sequence alignments of the ways that RrgA domain 4 has been split to create different protein coupling reagents. Numbering is based on PDB 2WW8.

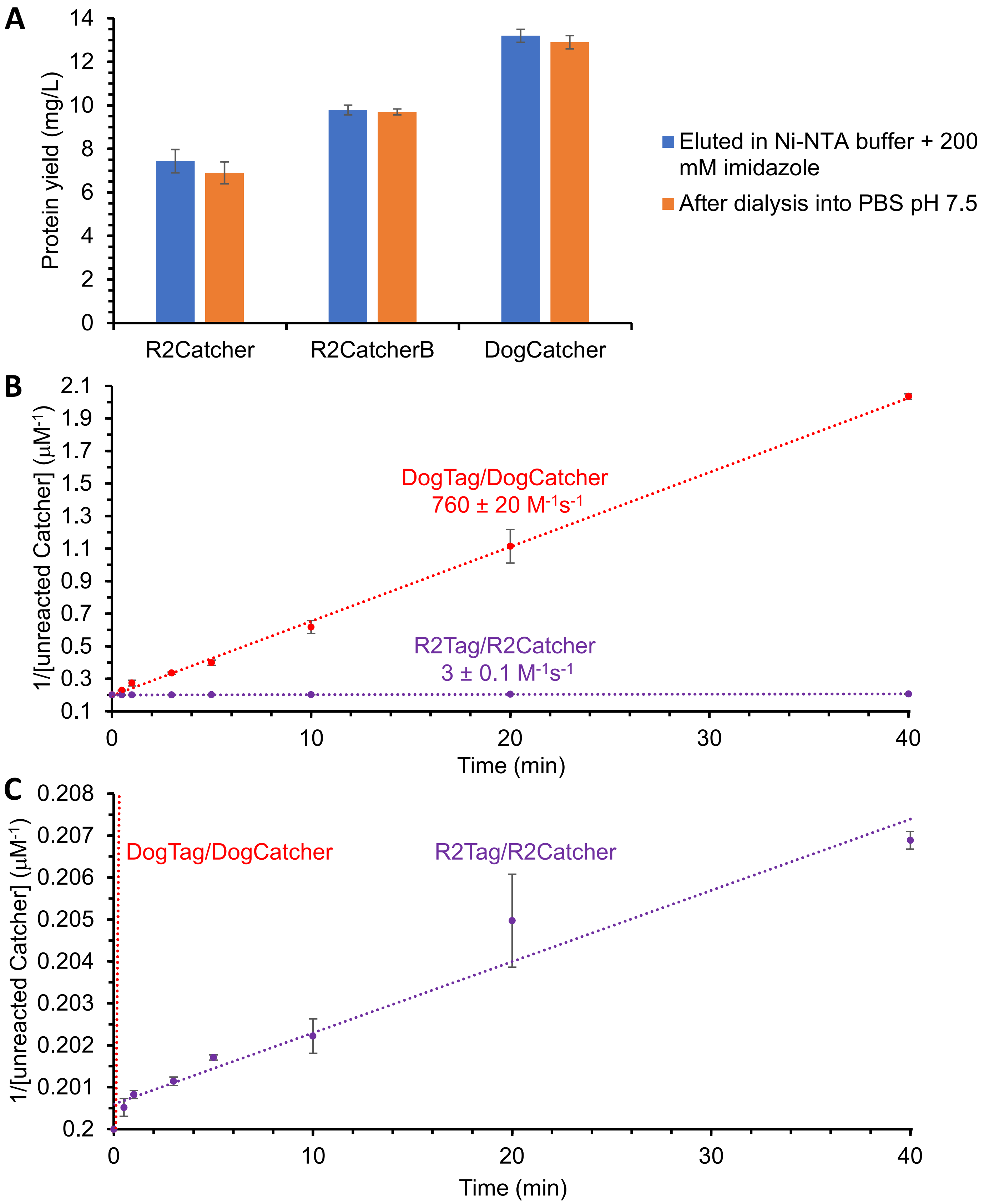

Figure S3: Comparison of solubility and reaction rate, related to Figures 1 and 5. (A) Yield of soluble protein for Catcher variants per liter of *E. coli* culture, determined after Ni-NTA elution (blue) or after dialysis (orange) (mean of duplicate with error bars signifying variation between measurements). **(B)** Time-course of reaction for DogTag/DogCatcher or R2Tag/R2Catcher. 5 μM AviTag-DogTag-MBP and 5 μM DogCatcher or 5 μM AviTag-R2Tag-MBP and 5 μM R2Catcher were incubated in PBS pH 7.5 at 25 °C, with quantification by SDS-PAGE/Coomassie (mean ± 1 s.d., n=3). Some error bars are too small to be visible. The resultant second-order rate constant is marked (mean ± 1 s.d., n=3). **(C)** Zoom of the y-axis from (B), to make the data clearer for R2Tag/R2Catcher.

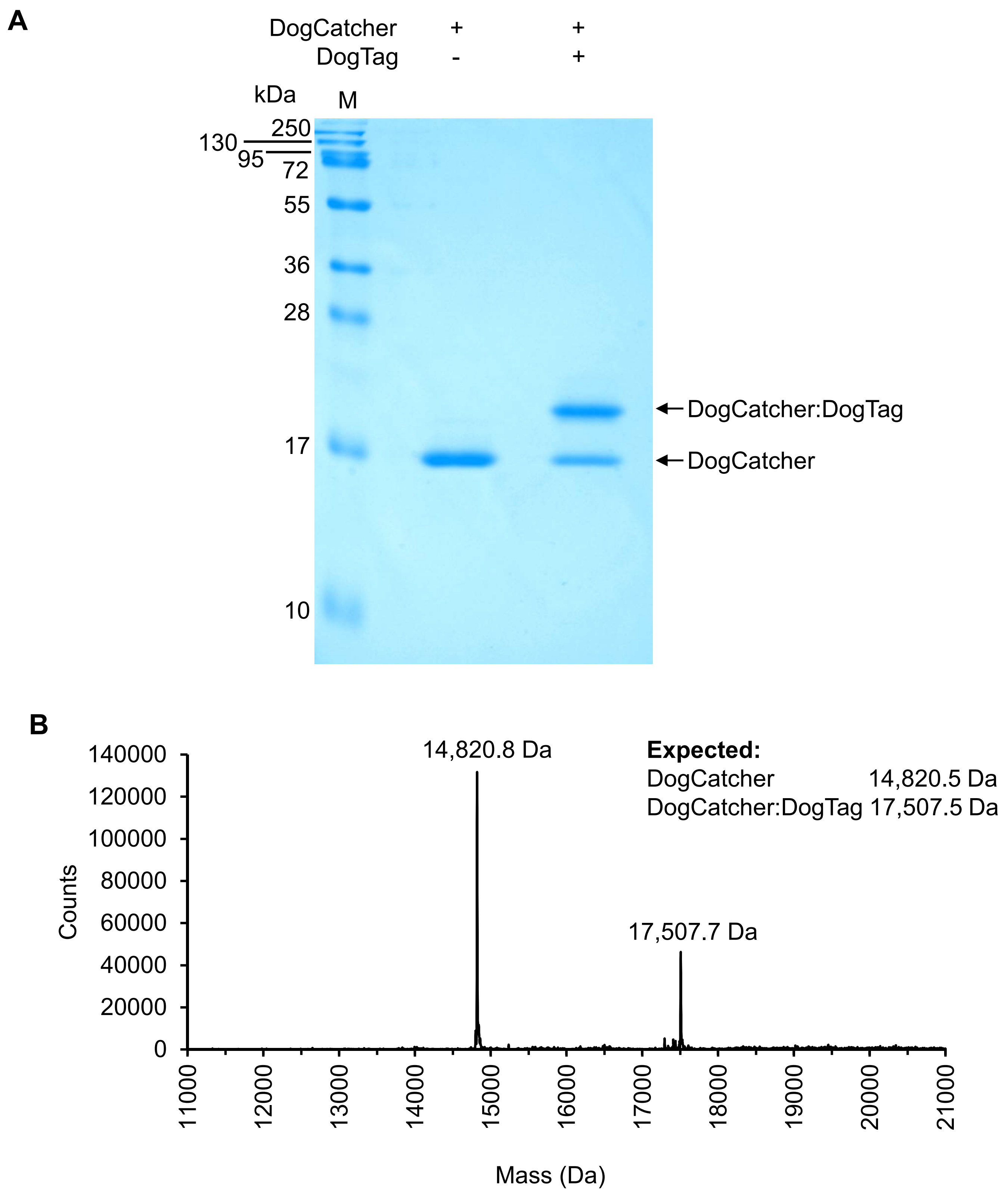

Figure S4: Electrospray ionization mass spectrometry validation of DogCatcher and reaction with DogTag, related to Figure 1. (A) SDS-PAGE with Coomassie staining, illustrating reaction of DogCatcher with DogTag peptide to approximately 50%, so that pre- and post-reacted DogCatcher can be compared. **(B)** Electrospray ionization mass spectrometry of DogCatcher and the reaction product of DogCatcher with DogTag peptide. Expected mass for DogTag peptide + DogCatcher = 17,524.5 Da minus 17 Da (NH_3 released upon isopeptide bond formation) = 17,507.5 Da.

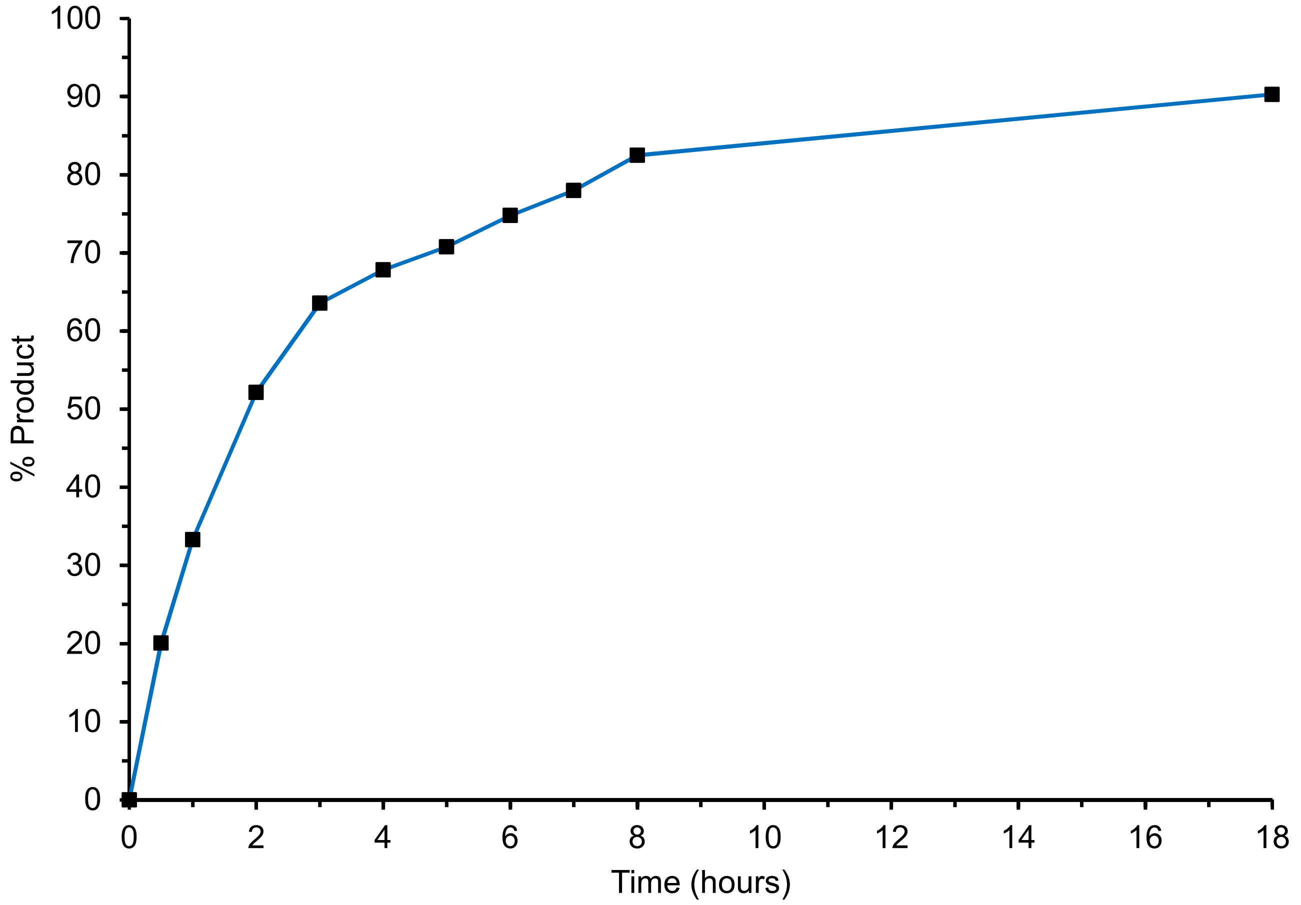

Figure S5: DogTag/DogCatcher reacted efficiently at nanomolar concentrations, related to Figure 3. Time-course of reaction for 100 nM HaloTag7SS-DogTag with 100 nM DogCatcher-sfGFP in PBS pH 7.5 + 0.2% (w/v) BSA at 25 ^{o}C, with quantification by SDS-PAGE/Coomassie and densitometry.

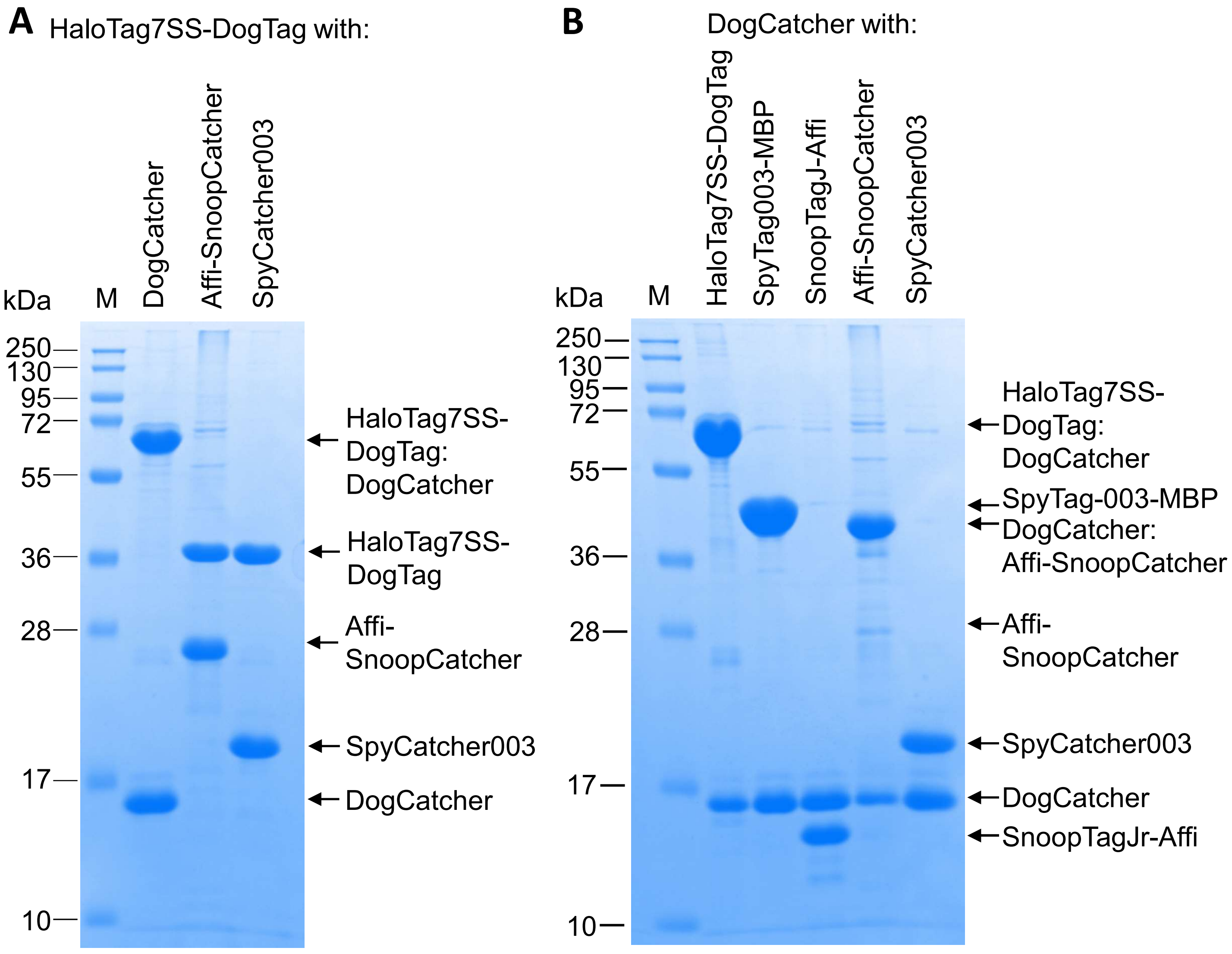

Figure S6: DogTag/DogCatcher orthogonality, as described in STAR Methods (Isopeptide Bond Formation Assays). (**A**) DogTag reacted with DogCatcher but not SnoopCatcher or SpyCatcher003. 15 μM DogCatcher, Affi-SnoopCatcher or SpyCatcher003 was incubated with 10 μM HaloTag7SS-DogTag for 24 h in PBS pH 7.5 at 25 °C, before SDS-PAGE with Coomassie staining. (**B**) DogCatcher reacted with DogTag and SnoopCatcher. 15 μM DogCatcher was incubated with 10 μM HaloTag7SS-DogTag, SpyTag003-MBP, SnoopTagJr-Affi, Affi-SnoopCatcher or SpyCatcher003 for 24 h in PBS pH 7.5 at 25 °C, before SDS-PAGE with Coomassie staining. M = molecular weight markers.

**Gre2p Variant**	**Specific activity ($\mu mol_{NAPDH}.min^{-1}.\mu mol_{protein}^{-1}$)**
WT	1,892 ± 294
SpyTag003 Loop A	2,391 ± 347
SpyTag003 Loop B	1,572 ± 372
SpyTag003 Loop C	1,814 ± 83
DogTag Loop A	3,087 ± 259
DogTag Loop B	3,268 ± 361
DogTag Loop C	1,484 ± 223

Table S1: Specific enzyme activities for Gre2p variants, related to Figure 6. Each Gre2p variant was incubated with isovaleraldehyde and NADPH in phosphate buffer at 25 ^{o}C and reaction was monitored spectrophotometrically (mean ± 1 s.d., n=3 biological replicates).
